# Nanogap-Engineered Core–Shell-Like Nanostructures for Comprehensive SERS Analysis

**DOI:** 10.1021/acsami.5c00716

**Published:** 2025-04-03

**Authors:** Mihai C. Suster, Aleksandra Szymańska, Tomasz J. Antosiewicz, Agata Królikowska, Piotr Wróbel

**Affiliations:** † Faculty of Physics, University of Warsaw, Pasteura 5, Warsaw 02-093, Poland; ‡ Faculty of Chemistry, University of Warsaw, Pasteura 1, Warsaw 02-093, Poland

**Keywords:** plasmonics, core−shell-like nanostructures, surface-enhanced Raman scattering, nanogap mode, nanosphere lithography, finite-difference time-domain, SERS substrates

## Abstract

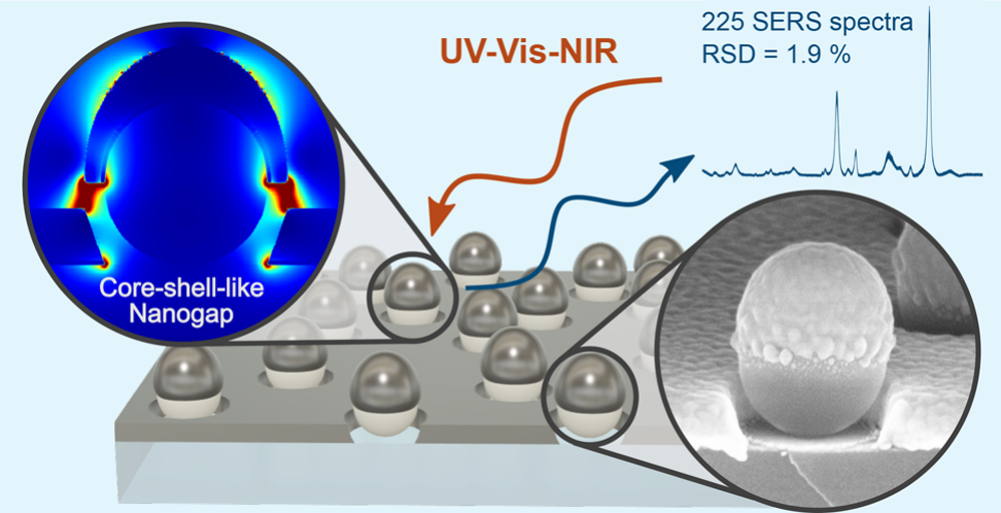

Development of fabrication protocols for large-area plasmonic nanostructures with sub-10 nm gaps with a spatially controlled distribution is critical for their real-world applications. In this work, we develop a simple, cleanroom-free protocol for the fabrication of macroscopic-sized plasmonic substrates (>6 cm^2^), featuring a tunable multiresonance optical response and light concentration in sub-10 nm gaps. Critically, these gaps are free to interact with the surrounding medium. This architecture consists of nonperiodically distributed dielectric nanospheres coated with a metal multilayer, forming semispherical core–shell-like nanostructures (CSLNs) surrounded by a planar film. The sub-10 nm gaps formed between metal caps and the planar film are easily tuned by adjusting fabrication parameters, such as multimetal layer thickness, composition, or nanosphere size and density. The excellent structural homogeneity, wide optical tunability, and extreme light confinement in the spatially controlled subwavelength nanogaps make CSLN-based substrates an ideal platform for comprehensive surface-enhanced Raman scattering (SERS) spectroscopy. This is proven through a combination of numerical modeling and iterative fabrication/characterization, leading to the optimized substrates showing cutting-edge spatial uniformity down to 1.9% determined as the relative standard deviation (RSD) of the SERS signal of *p*-mercaptobenzoic acid for 225 spectra over the 3600 μm^2^ area. High sensitivity is evidenced by an enhancement factor of ∼10.^6^ The proposed substrates also meet all other demanding criteria, including sufficient signal temporal stability (RSD <4%), high substrate-to-substrate reproducibility (<15%), and SERS activity toward three various analytes. The unique geometry and wide spectral tunability of the CSLN substrates will also be of great value for other plasmon-driven applications.

## Introduction

Interaction of light with metallic nanogap structures can result in extraordinary trapping and amplification of the electromagnetic (EM) field in regions down to the sub-10 nm scale due to the excitation of localized surface plasmon resonances (LSPRs). Such strong focusing of the EM field allows for studying new phenomena related to light–matter interactions, including enhanced absorption,^1^ nonlocality and ultrastrong coupling,^2,3^ quantum tunneling,^4^ and charge transfer plasmons.^5^ Plasmonic nanogap field enhancement also enables development of more efficient energy and light sources, as well as photodetectors,^3−5^ and provides an ideal platform for ultrasensitive detection of molecules,^6^ including surface-enhanced Raman scattering (SERS) spectroscopy^7,8^ with sensitivity reaching the single-molecule level.^9,10^

The most common approaches for fabricating plasmonic nanostructures featuring sub-10 nm nanogaps are based either on self-assembly of metal nanoparticles or on nanolithographic techniques, like electron and focused ion beam lithography.^11^ An example of the former is nanosphere lithography (NSL), which allows a relatively simple and large-area fabrication of nanogap structures. In NSL, dielectric nanospheres (DNSs) are assembled over a support to form a uniform hexagonally packed monolayer. Using this approach, along with a combination of ion etching and atomic layer deposition techniques, several architectures have been developed, featuring nanogaps in the few-nm regime. Examples include a metal film over nanosphere (MFON) structure,^7,12^ self-assembled nanorings with sub-10 nm gaps,^13^ and nanoforests with gold nanoparticles deposited on top of a hexagonal array of nanopillars.^14^ In the latter case, a metal-thickness-dependent nanogap is formed between the nanoparticle and the discontinuous gold film deposited in the interpillar voids on the substrate. However, the aforementioned approaches either suffer from a lack of repeatability and insufficient resolution of the nanogap size—not reaching below 10 nm—or produce structures featuring plasmonic hot spots inaccessible to the external environment and exhibiting a nonspecific broad optical response. Due to these limitations, there is still a growing demand for a simple, low-cost, and high-throughput technology capable of fabricating large-area plasmonic nanostructures with easy-to-access gap sizes below 10 nm, offering a rich and tunable optical response.

Development of new nanofabrication procedures is especially desired in the field of SERS spectroscopy, which can now be routinely used to identify and quantify chemicals, even with single-molecule sensitivity.^9,10^ Due to its high sensitivity and flexibility of design, SERS is widely used in a variety of areas including analytical,^15^ imaging,^16^ environmental,^17^ pharmaceutical,^18^ medical,^16^ chemical,^15,17^ biological,^16^ and security-related applications.^19^ SERS’ usefulness is enabled by enhancing the otherwise very weak Raman scattering signal using two distinct mechanisms: EM field enhancement and chemical effect (mainly due to charge transfer resonances).^9,20^ The dominant EM contribution to the total amplification of the Raman signal is possible by the use of noble metal plasmonic nanostructures, which is why in recent years, they have become somewhat synonymous to SERS.

Although SERS has been recognized for over 50 years as an excellent scientific tool, reproducibility of the results remains a challenge.^21^ Indeed, this handicap is to be a major limitation to the widespread use of SERS outside the laboratory, which triggered the establishment of rigorous standards for evaluating SERS-active plasmonic substrates.^22,23^ Specifically, five main criteria for a high-quality SERS-active substrate were formulated:^22^ (1) high spatial reproducibility defined as less than 20% spot-to-spot variation of the SERS signal over 10 mm^2^; (2) high substrate-to-substrate reproducibility, validated by less than 20% SERS signal variation over 10 substrates of the same type; (3) sufficient temporal stability allowing for a maximum 20% SERS signal variation measured weekly for a month; (4) high sensitivity with the recommended enhancement factor exceeding 10^5^ over an area of at least 7500 μm^2^; and (5) documented SERS activity toward three analytes not exhibiting surface-enhanced resonance Raman scattering (SERRS) established for neutral, positively charged, and negatively charged adsorbate molecules. Despite many efforts dedicated to successful fulfillment of these demanding standards, the task remains unresolved.^9^ Critically, many works emphasize optimization of the substrates in just one of the aforementioned areas without considering the others.

Herein, we report the development of remarkably spatially uniform plasmonic substrates of macroscopic size (>6 cm^2^) that meet all five criteria described above as vital for comprehensive SERS analysis. The proposed design offers a simple, scalable, and easily tailorable fabrication method leveraging straightforward metallization of a solid support decorated with nonperiodically distributed DNSs. This new nanoarchitecture exhibits a core–shell-like behavior and features easily accessible nanogaps of desired size in the sub-10 nm range, as well as multiple plasmonic resonances tunable via simple adjustment of a few fabrication parameters. Due to extreme light confinement leading to enormous local enhancement of the EM field, such nanogap-engineered plasmonic substrates facilitate a strong and long-term stable enhancement for SERS spectroscopy, an exceptionally spatially uniform surface enhancement over an area exceeding 6 cm^2^, as demonstrated for the Raman probe of *p*-mercaptobenzoic acid. This development paves the way for plasmonic platforms with customizable nanoarchitectures based on tunable core–shell-like nanostructures, suitable for various applications requiring both a tunable, multimodal optical response and both point-to-point and sample-to-sample reproducibility.

## Results and Discussion

### Fabrication of Core–Shell-Like Nanostructures (CSLNs)

**Figure 1:**
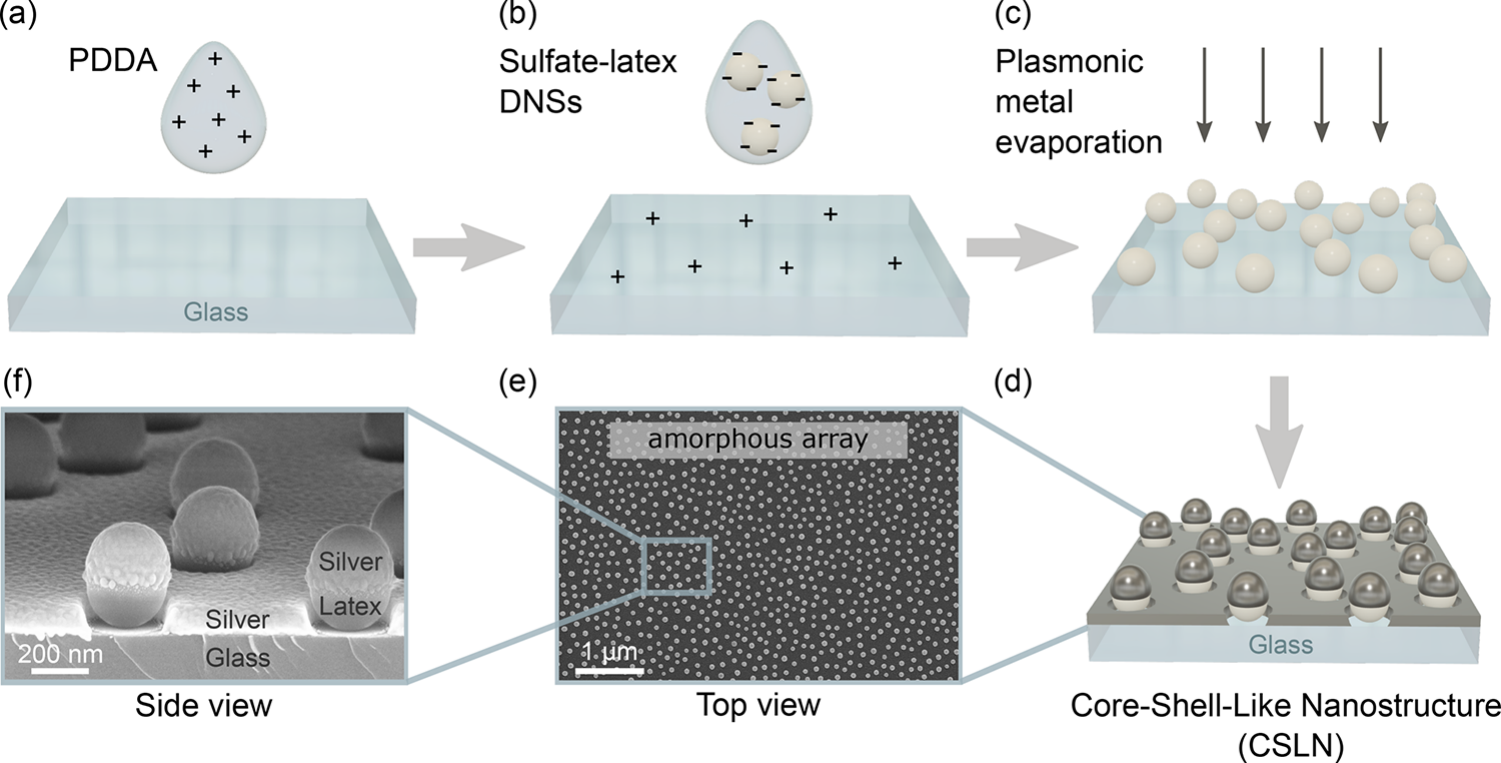
Schematic illustration of the fabrication process of an amorphous array of CSLNs: (a) electrostatic attraction between a layer of positively charged PDDA polymer initially deposited on a glass support and (b) following drop-cast negatively charged sulfate latex DNSs results in an amorphous array of DNSs. (c) The array is then covered by a plasmonic metal using the PVD technique, (d) leading to the formation of the CSLN geometry featuring nanogaps between the metal caps and the planar metal layer formed on the glass support. (e) Large-scale SEM image (top view) of a global distribution of CSLNs within an amorphous array and (f) SEM image (side view) showing individual CSLNs with recognizable metal caps and nanogaps.

The key steps of the proposed protocol of fabricating CSLNs are schematically shown in Figure 1. The fabrication process is based on the NSL framework.^24−26^ First, negatively charged sulfate latex DNSs are deposited on a solid glass support covered initially with a positively charged thin layer of poly(diallyldimethylammonium chloride) (PDDA) (see Figure 1a,b). Electrostatic forces between same- and opposite-sign electric charges ensure surface trapping of particles and their separation,^25^ as well as a short-range correlated (amorphous) distribution of the DNSs.^27^ Next, directional physical vapor deposition (PVD) of a germanium wetting layer and a thin plasmonic metal film (Figure 1c,d) creates a cap over each nanosphere separated from the planar metal layer deposited between the DNSs. This results in the formation of CSLNs with uniform nanogaps between the caps and the planar film (see Figure 1d for the schematic representation and Figure 1f for the actual geometry imaged by using scanning electron microscopy (SEM)). The size of these nanogaps can be easily controlled by adjusting the evaporated film thickness and the diameter of the DNSs. This approach, while being a modification compared to conventional NSL methods, remains straightforward and allows to achieve a unique geometry with features in the sub-10 nm regime.

As presented in the top-down SEM image in Figure 1e, the proposed fabrication protocol results in homogeneous coverage with an amorphous array of nanospheres with no sign of aggregation. Uncontrolled aggregation is a common concern in bottom-up approaches of nanostructure assembly onto a solid support, which translates into an inhomogeneity of the resulting geometrical arrangement. Particularly in SERS analysis, this leads to spectral irreproducibility related to random formation of highly localized regions of intense EM fields, so-called “hot spots”. In our approach, suppression of oligomerization is ensured by strong nanosphere-PDDA polymer electrostatic attraction and mutual repulsion between the same-sign charged latex nanospheres, which jointly overcome interparticle van der Waals/Casimir attraction (see SEM images in Figure S1 in the Supporting Information (SI) and the discussion in Section S1 therein).

The SEM image in Figure 1f shows a side view of a typical nanostructure obtained herein. The upper half of the DNS is covered with a metal cap, the maximum thickness of which at the very top of the sphere is equal to the nominal thickness of the planar layer on the substrate. The thickness on the side of the DNS decreases on average to less than 30% of the nominally evaporated value (see Figure S2 to examine the details). Such a geometry is ascribed to an interplay of two coexisting effects. The first one is related to different wettability and varying metal atom flux normal to the local particle surface, resulting from the curvature of the DNS. The second effect is due to the shadow cast by the nanosphere, which determines the diameter of the aperture within the planar metal film deposited on the glass around the DNS (see SEM images in Figure S2 for details). This shaded area, inaccessible to metal atoms during evaporation, increases with the thickness of the metal layer on the side of the sphere. Hence, the aperture walls tilt away from the DNS, as can be seen in Figure 1f.

Continuity of both the cap and the bottom metal layer is achieved by evaporating a 1.5 nm-thick Ge film, which serves as a wetting layer to prevent island-growth of silver.^28^ This subtle step in the fabrication protocol leads to major changes of the nanostructure’s geometry: in the absence of Ge, the wettability of DNSs is very low, and therefore, the evaporated silver would form one ill-defined bulk nanoparticle on top of a single DNS. The substrate uniformity in such a case would be very low, as each DNS would be covered with a metal nanoparticle of different shape and size. An increase in the wettability allows silver to create a well-adhering cap around the upper half of each DNS (compare respective SEM images and absorbance spectra in Figure S3 in the Supporting Information and the discussion therein, Section S1) with the final structure resembling a core–shell geometry. Germanium also smooths out the planar metal layer, resulting in a well-defined toroidally shaped nanogap between the aperture edge and the cap (see SEM images in Figure S2). Increasing the thickness of the metal film naturally leads to closing of the nanogap, which formally is expected at the evaporated thickness corresponding to the radius of the DNS. However, due to the directionality of the evaporation process and the aforementioned shadow effect, the aperture size increases upon metal layer growth, and thus the gap is still present for metal film thicknesses exceeding the DNS radius size. Table S1 shows a summary and comparison of the proposed approach with other state-of-the-art nanofabrication techniques used for the development of high-performance SERS substrates like electron beam lithography,^29^ bottom-up synthesis of nanosphere-on-mirror,^30^ tip-based nanofabrication,^31^ or NSL.^32^

### FDTD Calculations

FDTD calculations were performed to predict and elucidate the optical response of the CSLNs. Using the nominal sizes of nanospheres, the amount of deposited metal, and particle surface density determined from SEM images, we developed a numerical model of the fabricated nanostructures. While most parameters describing an individual particle can be reliably represented in simulations, the disordered nature of the CSLN metasurfaces can only be treated in an approximate way. The lack of ordering means that to properly account for phase relations between the multiply scattered and incident fields, one would need to model a few thousand particles, including interactions with the substrate and perforated thin silver film, which is not feasible for practical purposes, especially in FDTD modeling. An often used, yet approximate, approach is to employ periodic boundary conditions to qualitatively capture interparticle coupling. However, this may yield results that are qualitatively similar to an experimental realization with an amorphous array, especially if multiple resonances are present within the spectral region of interest. Consequently, when modeling disordered arrays, some discrepancies are expected; however, the overall trends can be still simulated appropriately.^33^

**Figure 2:**
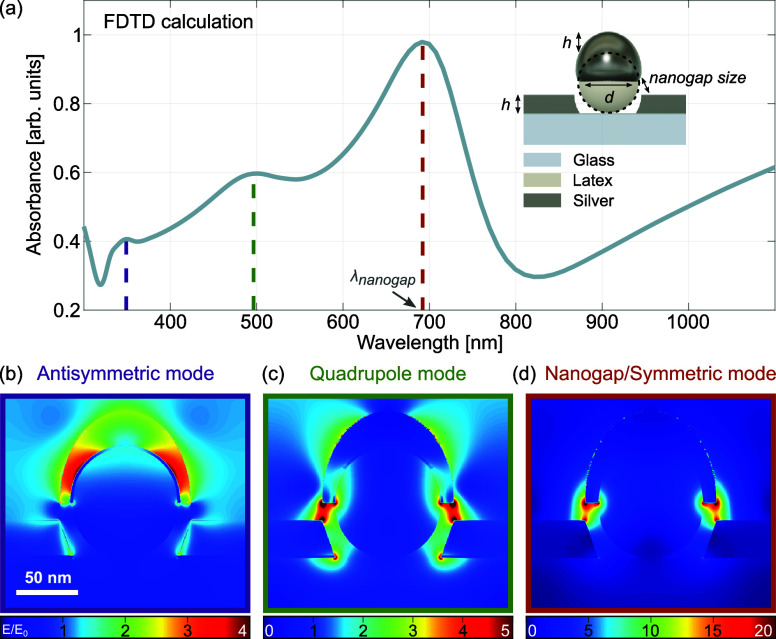
(a) FDTD-calculated absorbance spectrum of CSLN with a 60 nm dielectric core diameter (*d*) and a Ag layer thickness (*h*) of 20 nm, resulting in a nanogap size of 8.5 nm (see the model geometry on the right). (b–d) Electric field distributions for the structure depicted in (a) corresponding to the plasmonic modes calculated at the resonant wavelengths indicated by the color-coded dashed lines in the plot shown in (a). The types of plasmonic modes are given in the legend.

Figure 2a shows the absorbance spectrum calculated for a nanostructure with DNSs of 60 nm in diameter (*d*), a 1.5 nm-thick Ge layer, and a Ag nominal thickness (*h*) of 20 nm, which results in a gap size of 8.5 nm (cf. the scheme in the inset of Figure 2a for the model of a single simulated nanostructure). The thickness of the cap at the side of the DNS is 30% of that on the top, according to the experimental data (cf. SEM images and parameters in Figure S2). The simulated absorbance spectrum exhibits three distinctive plasmon resonances associated with the electric field distributions depicted in Figure 2b–d, where the colors of the frames match those of the dashed lines in Figure 2a, indicating the respective spectral positions. The most intense maximum at ∼700 nm (marked as λ_nanogap_ in Figure 2a) corresponds to a symmetric nanogap mode formed between the metal cap and the edge of the aperture within the planar metal layer (Figure 2d). The field amplitude associated with the resonance at ∼500 nm exhibits a higher-order, quasi-quadrupolar nature (Figure 2c). However, it shows notable deviations from the classical quadrupole mode observed for the bulk metal nanospheres due to the coupling of the two bottom lobes with the nanogap mode. The enhanced EM field surrounds the nanogap, ensuring that the largest amplitude is located therein. The electric field distribution for the resonance peak at ∼385 nm exhibits a maximum at the DNS/metal cap interface (Figure 2b) and strongly resembles the antisymmetric mode characteristic for core–shell nanoparticles.

**Figure 3:**
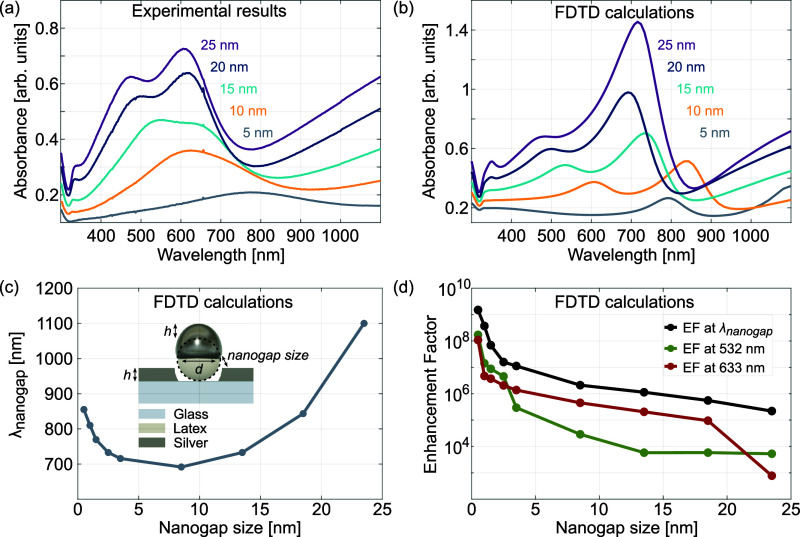
Absorbance spectra of CSLNs based on DNSs with a 60 nm dielectric core diameter (*d*) and a Ag layer thickness (*h*) varying from 5 to 25 nm: (a) as measured in the experiment and (b) as calculated via the FDTD method. (c) FDTD-calculated relationship between the wavelength of the nanogap resonance and silver layer thickness (calculated for nine values of *h* and *d* = 60 nm for model geometry in the inset). (d) Semi-log plot of FDTD-calculated values of the enhancement factor (EF) for varying gap sizes at the nanogap resonance wavelength and at Raman excitation wavelengths of 532 and 633 nm (calculated in each case for nine values of nanogap size and *d* = 60 nm). The size of the nanogap for (c) and (d) is expressed in simulations as *d*/2 – *h* – *h*_Ge_, where *h*_Ge_ is the thickness of Ge (1.5 nm), while the lines connecting the points are a guide to the eye.

The calculations indicate that the CSLNs exhibit a response reminiscent of core–shell plasmonic particles, although modified by coupling to the aperture. The response of a typical core–shell plasmonic nanosphere stems from coupling of two dipolar modes, with one being that of a metal sphere and the other of a void in metal. This interaction yields a symmetric dipole mode across the whole particle and a higher-order antisymmetric dipole resonance, the spectral separation of which depends on the metal shell thickness. As the shell/core ratio increases (at a constant core size), the spectral separation of the two resonances diminishes, with the higher-order mode decreasing in amplitude and the remaining single resonance acquiring the characteristic properties of a dipole mode of a bulk metal nanoparticle.^34^ By observing such a trend for CSLNs, one could confirm that the metal-capped DNSs located in apertures exhibit a behavior similar to that of fully coated core–shell nanoparticles. Therefore, the influence of the metal layer thickness on the optical response of the CSLN structure was calculated and compared with the experimental data. Figure 3 compares the absorbance of CSLNs based on a 60 nm DNS coated with a silver layer with thickness varying from 5 to 25 nm as measured in the experiment (Figure 3a) and calculated via the FDTD method (Figure 3b). Both sets of curves show a similar characteristic trend. Increasing the thickness of the silver layer leads to a blue shift of the energies of both the nanogap and quadrupole modes, while the resonance in the UV range is shifted toward longer wavelengths. Apart from the overall increase of the absorbance due to the presence of a larger amount of lossy material, the amplitude of the plasmonic resonances is substantially improved with increasing silver thickness, which is likely related to diminishing nonlocal effects and reduced electron scattering in thicker metal films.

Overall, the optical response of the analyzed CSLN is consistent with that of a conventional core–shell nanosphere, whose FDTD-calculated EM properties are presented in Figure S4 (extinction, scattering, and absorption cross sections and electric field distribution for the 60 nm-diameter dielectric core and the 20 nm Ag shell) and Figure S5 (extinction cross section as a function of the silver layer thickness for 60 nm DNS), further discussed in Section S2 of the Supporting Information.

The main features of the experimental absorption data and the effect of the thickness of the plasmonic metal film are well reproduced by FDTD simulations (cf. Figure 3a,b).

Discrepancies between experimental and numerical results in terms of the exact positions of resonances and their widths and amplitudes are typical when considering the approximations, especially for the disordered nature of the arrays used in the idealized model. It is worth stressing that the simulations do not take into account any imperfections observed in the experiment, such as dispersion of the nanosphere size and surface roughness, as well as the tendency of silver to form granular features at the rim of the cap rather than a solid and well-defined edge. Moreover, due to the directionality of the evaporation process, substrates placed off the rotation axis may exhibit slight asymmetry of the metal cap, leading to a modified spectral response of the CSLNs. Finally, and in principle most importantly, the amorphous and thus disordered nature of the nanoparticle array means that in most modeling techniques, it is not possible to account for the exact phase shift between scattered and incident light, especially for dense arrays exhibiting multiple resonances. Reproducing the correct phase is necessary, as the interaction modifies the single-particle resonances affecting all key spectral parameters including peak position, width, and amplitude.^33^ This modification is nonmonotonous with a minimum interparticle distance in an amorphous array and typically cannot be correctly reproduced using simple periodic boundary conditions. To properly model a single CSLN particle, including its few-nanometer-sized features and complex geometry, FDTD is one of the few methods of choice. However, FDTD cannot be used to calculate the optical response of a few thousand particles to account for the random character of the interaction. Hence, a qualitative agreement with experiment of the shifts of the exact position, amplitude, and width of the resonances is expected,^33^ but these modifications will be different for the particular resonances exhibited by the CSLN particle, making a precise quantitative agreement with experiment practically impossible. Additionally, absorbance measurements were performed with an incoherent light source, which decreases the resonance contrast in comparison to simulations assuming a coherent linearly polarized plane wave. Further discussion on the discrepancies between theory and experiment can be found in Section S2, along with Figure S6, which summarizes the influence of the most critical parameters in the FDTD model on the nanogap resonance position (λ_nanogap_) and its peak amplitude. However, despite these caveats, the fundamental properties of CSLNs are satisfactorily explained by the FDTD modeling.

Figure 3c shows the FDTD-calculated changes in the spectral position of the plasmon resonance corresponding to the mode due to nanogaps of varying size for 60 nm DNSs. For the 8.5–23.5 nm range of the nanogap size, this resonance mode exhibits a blue shift as the size of the nanogap decreases (and thus the thickness of the silver film increases), which is typical of the symmetric mode in core–shell nanoparticles. In contrast, for a nanogap size less than 8.5 nm, we observe a red shift and further increase of the discussed resonance peak’s amplitude with decreasing gap size (increasing metal thickness), as can also be seen in simulated absorbance spectra in Figure 3b. The origin of the resonance red shift is strong coupling between the plasmons excited at the cap and those at the aperture edge, which is a typical optical response for the nanogap structures.^35,36^ In other words, as the gap size decreases, two different behaviors of this resonance peak occur, confirming its dual origin predicted by FDTD. For gaps much larger than 10 nm, the resonance undergoes a blue shift like in a typical core–shell nanostructure, and for gaps smaller than 10 nm, it red-shifts like for a standard nanogap mode (as seen in Figure 3b,c). This leads also to a large EM field enhancement that can be expressed as an enhancement factor (EF), calculated as EF ∼ |*E*/*E*_0_|,^4^ where *E* and *E*_0_ are amplitudes of the electric field of the resonant plasmon mode (here: nanogap mode) and the incident light beam, respectively, assuming that the incident and inelastically scattered photons are of similar energy. The semi-log plot in Figure 3d shows the numerically calculated EFs as a function of the nanogap size for the CSLNs architecture at λ_nanogap_ (nanogap resonance wavelength), as well as 532 and 633 nm wavelengths, used as excitation lines in the following SERS experiments. For nanogap sizes above 5 nm, the EF is in the range of 10,^6^ while for smaller gaps, it increases to 10^7^ and reaches 10^9^ for the gap sizes below 1 nm (Figure 3d). Use of photons with wavelengths within the plasmon resonance peak, but not matching exactly its maximum, results in a decrease of EF by only one order of magnitude, which means that an EF of around 10^8^ can be reachable in practice.

### Optical Response and Tuning

Small changes in the easily adjustable parameters of the CSLN fabrication protocol can result in drastic or minimal modifications in the plasmonic properties of the final structure, depending on the modified parameter. This makes the CSLN a very convenient and practical platform, as its optical features can be easily tailored specifically to meet the desired requirements of a particular application. The process of tuning the spectral position of one or more of the available plasmon resonances can be divided into two stages: coarse and fine spectral tuning. Coarse tuning involves modifying the fabrication parameters that induce major spectral changes by shifting the resonance wavelengths by many tens or even hundreds of nanometers. Then, the position of the resonances can be fine-tuned with resolution reaching single nanometers by adjusting other, much less impactful parameters that only slightly change the resonant energy while preserving the overall optical response of the entire photonic system.

**Figure 4:**
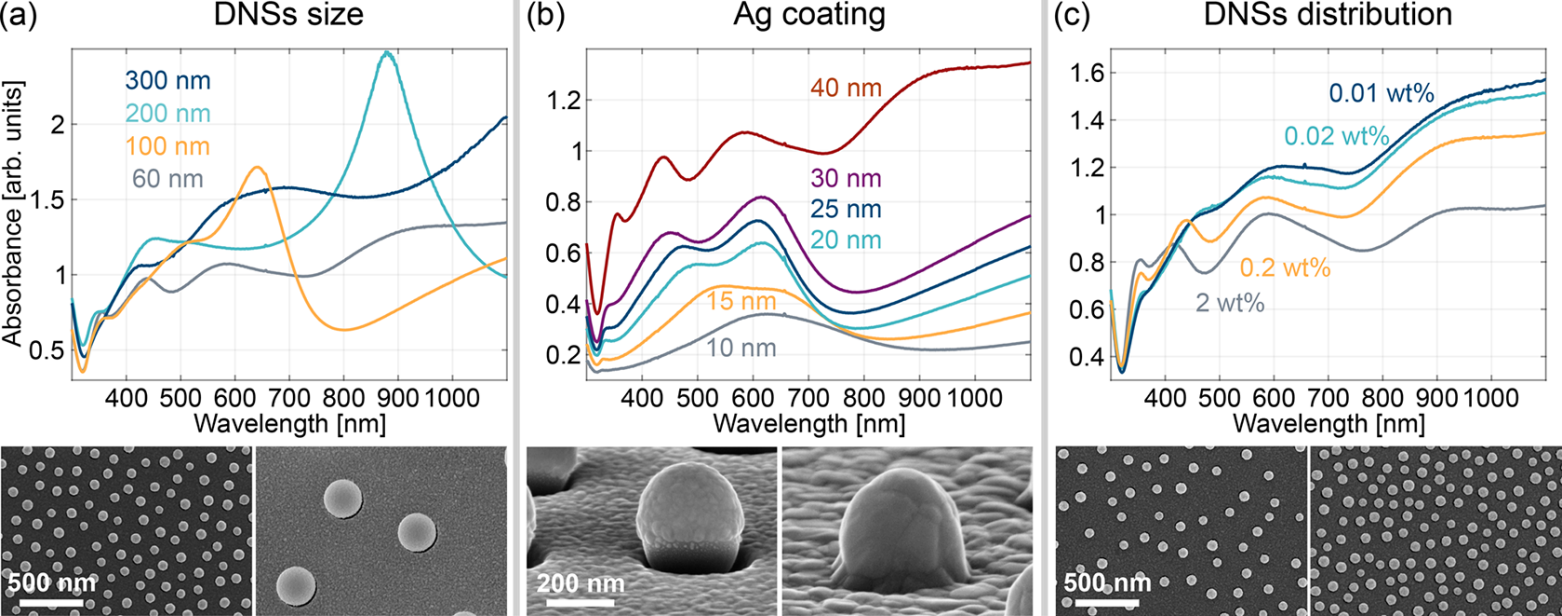
Demonstration of rough and fine spectral tuning capabilities within the CSLN architecture: (a) rough tuning by using DNSs of different diameters (fabrication parameters: 0.2 wt % suspension of 60–300 nm DNSs, metal multilayer: 1.5 nm Ge and 40 nm Ag); (b) fine-tuning by varying the silver layer thickness (0.2 wt % suspension of 60 nm DNSs, 1.5 nm Ge, 10–40 nm Ag); (c) ultrafine tuning by changing the mean distance between the DNSs, controlled by their bulk concentration (0.01–2 wt % suspension of 60 nm DNSs, 1.5 nm Ge, 40 nm Ag). The values of (a) the DNS diameter, (b) the nominal thickness of the silver layer, and (c) the bulk concentration of DNS suspension are given in the legend. Top row: absorbance spectra showing the spectral tuning characteristics of a given fabrication parameter. Bottom row: SEM images illustrate pronounced effects of a given parameter on the morphology of the CSLN.

There are about 10 relevant parameters that can be adjusted during the CSLN fabrication process to affect the absorbance spectra, enabling shifts in the energy of a specific plasmonic mode of the substrates. Representative examples of spectral tuning using selected single experimental factors, along with corresponding SEM images showing the resulting structural differences in CSLNs, are presented in Figure 4. First, coarse spectral tuning is achieved, e.g., by using DNSs of different diameters (Figure 4a, top row). This change also affects the overall topography of CSLNs in terms of the particle density (cf. SEM images for 60 and 300 nm DNSs in Figure 4a, bottom row) and the nanogap size and its optical response for a given Ag thickness. For example, changing the diameter of the DNSs from 100 to 200 nm (i.e., by 100%) spectrally shifts the gap mode by approximately 250 nm (from 640 to 890 nm), as can be seen in Figure 4a, top row. The two other plasmonic modes, i.e., the quadrupole and antisymmetric modes, are also red-shifted with an increasing diameter of the DNSs, but to a much lesser extent.

Next, fine-tuning can be realized, *e.g*., by varying the thickness of the metal coating. Figure 4b (top row) illustrates that as the silver layer thickness increases, the quadrupole peak mode undergoes a blue shift. For example, evaporating 30 nm of Ag instead of 15 nm (i.e., changing *h* by 100%) shifts the quadrupole mode by ca. 70 nm (from 510 to 440 nm). An analogous blue shift for an increasing metal shell thickness accompanied by a simultaneous narrowing of the peak width is observed for the nanogap plasmonic mode. However, one has to keep in mind that an additional effect of an increasing metal thickness is a gradual closing of the nanogap (see SEM images for a 300 nm-diameter DNS coated with 100 and 300 nm of Ag shown in Figure 4b, bottom row). Finally, the antisymmetric mode shifts consistently toward the red as the Ag layer is growing, and its intensity increases significantly when the nominal shell thickness exceeds half of the DNS diameter (cf. absorption spectra for *h* = 30 and 40 nm for 60 nm DNS in Figure 4b, top row).

Lastly, ultrafine tuning can be accomplished, e.g., by heavily diluting the suspension of DNSs, which results in an increased mean interparticle distance (see SEM images for 2 and 0.01 wt % for 60 nm DNSs in Figure 4c, bottom row). This allows for subtle resonance peak shifting (Figure 4c, top row) due to modification of interparticle coupling.^33^ All three observed resonance peaks are red-shifted as the average distance between the DNSs increases with the decreased concentration of the suspension, although not necessarily with equal magnitude nor monotonously across the whole examined parameter range, which is caused by different phase delays between each individual mode.^37^ The total substrate coverage by DNS decreases with lowering of the bulk concentration; therefore, the contrast of all the peaks deteriorates as the overall structure acquires a reflective character with fewer DNSs dispersed on the substrate. The antisymmetric mode is the least affected, since a change of DNS concentration from 0.02 to 0.01 wt % red-shifts the quadrupole mode by less than 10 nm. Hence, DNS concentration is a very convenient parameter for ultrafine tuning of the optical response because a significant, and thus easy to precisely control, dilution causes subtle (even a single nanometer) changes in the position of resonances.

Using a combination of only these three parameters already enables generation and positioning of plasmonic resonances across the whole visible (Vis) and near-infrared (NIR) spectral range. If needed, further tuning can be performed, e.g., by deposition of the CSLNs on a different support, using DNSs with a changed refractive index, or evaporating a more sophisticated multilayer consisting of a number of materials (metals and/or dielectrics) and many more. The main reason for choosing these three parameters in the tuning procedure described above is that they can be easily adjusted in a continuous manner. The DNSs can be synthesized in any size in the range from around 40 nm up to a few μm, the plasmonic metal film can be evaporated with a sub-nm-thickness precision, and the interparticle distance can be controlled by heavily diluting the DNS suspension. This provides an elegant and straightforward strategy for tailoring the plasmonic properties of CSLNs by perfectly matching a target wavelength imposed by a specific application. Moreover, the presence of three plasmonic resonances makes these substrates particularly useful for SERS spectroscopy, since it is possible to tune the peaks simultaneously (as well as individually) to the energies of the excitation line and the Stokes bands of the molecules, thus enabling efficient, double- or even multi-resonance SERS measurements.^38,39^

Having established the physical justification of the optical modes of the CSLN nanostructure, which yield the field enhancement necessary for the SERS effect, we consider one final aspect. While silver is the superior metal for large field enhancements in visible-light excited plasmonic applications, it does not offer good chemical stability under all conditions. Hence, for practical applications, it is recommended to consider passivation layers that will protect silver against corrosion.^40^ However, the use of additional dielectric layers on top of a plasmonic metal will substantially decrease the field intensity experienced by molecules, lowering the EF significantly. Hence, gold, due to its greater chemical inertness and biocompatibility, is a better choice than silver in (bio)sensing, despite the larger losses from interband transitions extending far into the visible.^41^ Thus, we leverage the best parameters of both materials by using the low-loss silver as a bottom layer and coating it with 5 nm of Au. This should ensure good optical properties while significantly enhancing the stability of the experiment and protecting the nanostructure from the harmful effects of harsh solvents. As plotted in Figure S7, an additional 5 nm Au layer on top of 20 nm of Ag for 60 nm DNSs predictably shifts the nanogap resonance to the position similar to 25 nm of Ag. This shows that the overall evaporated metal thickness and thus the resulting nanogap size govern the position of the nanogap resonance. On the other hand, the addition of 5 nm Au leads to a diminished quadrupole mode due to the interband transition in gold occurring for wavelengths below 516 nm.^42^ Thus, bearing in mind the minor effect of gold on the essential features of surface plasmon resonances (see Figure S7 and discussion in Section S3 of the Supporting Information), we employ only CSLN nanostructures with a variable amount of silver, which is capped by 5 nm of Au in the SERS characterization that follows.

### SERS Performance of CSLN Substrates

We now turn to the examination of the SERS performance of the proposed CSLN plasmonic platform with respect to the five criteria that determine a high-quality SERS substrate, as specified in the Introduction section. Validation of these demanding requirements is carried out via comprehensive SERS studies, which confirm the fulfillment of all five conditions by the developed substrate. For a succinct summary of the outcomes of this study, we refer to Table 1, while below we introduce the experimental details followed by a presentation and discussion of the results.

**Table 1:** Verification if CSLNs Satisfy the Criteria of a High-Quality SERS Substrate^22,23^^a^

**criterion type**	**required**	**observed for CSLNs**	**evaluation**
#1 high spatial reproducibility	less than 20% spot-to-spot variation of SERS signal over 10 mm^2^	top result: RSD of 2.7% for the most spatially uniform substrates (2 wt %) and RSD of 2.8% for the optimized substrates, measured at 675 points over approximately 140 mm^2^ (see Figures 6g and 7a,b) using a 532 nm laser. RSD of 1.9% for 225 spectra and *ca.* 3600 μm^2^ area (for 2 wt %).	cutting-edge result
		less than 20% for all the substrates except the largest DNS size (300 nm) and the lowest DNS concentration (0.01 wt %) for 632.8 nm laser (see Figure 6g–i)	more than satisfied
#2 high substrate-to-substrate reproducibility	less than 20% SERS signal variation over 10 substrates of the same type	RSD of 14.7% for 21 spots (225 spectra from *ca.* 3600 μm^2^ rectangular area for each spot) distributed across various fragments originating from macroscopically cut sections of six optimized substrates of the same type fabricated within 5 months (see Figure S15)	satisfied
#3 sufficient temporal stability	20% or less SERS signal variation, measured weekly for a month	deviation from the original SERS intensity of 3.6% for the optimized substrate without Au and 18.7% for the optimized substrate with Au, measured once after 4 months from fabrication (see Figure S14)	more than satisfied
#4 high sensitivity	values of EF exceeding 10^5^ over an area of at least 7500 μm^2^	EF typically above 0.33 × 10^6^ for optimized substrate with Au and up to 1.5 × 10^6^ for optimal substrate without Au, determined over an area of approximately 17 000 μm^2^ (see Table 2)	satisfied
#5 SERS activity toward three analytes	SERS signal for three chemicals not exhibiting SERRS effect: with positively charged, neutral, and negatively charged adsorbate molecules (for each case)	SERS activity for the optimal substrates documented toward pMBA (anionic/neutral), Pyr (cationic/neutral), and MO (methyl orange; anionic/neutral) under nonresonant conditions (see Figure S16)	satisfied

a The assessment involves the SERS band of pMBA at 1585 cm^–1^ for optimized substrates fabricated with 0.02 wt % suspension of 60 nm DNSs, 1.5 nm Ge, 40 nm Ag, and 5 nm Au, unless stated otherwise.

SERS validation of the CSLN substrates is performed using *p*-mercaptobenzoic acid (pMBA; see the inset in Figure S8 for the molecular formula), which is also known as 4-mercaptobenzoic acid (4-MBA) and can be considered as a representative of a neutral and/or anionic molecule, depending on the conditions of the experiment. The SERS signature of pMBA is well recognized since it was first reported by Michota and Bukowska.^43^ The monolayer of pMBA is formed through self-assembly, where the molecules are firmly attached to the surface by covalent bonding between the metal (Au and Au) and sulfur. In addition, pMBA exhibits a large Raman scattering cross section typical of benzene derivatives and its SERS signal is sensitive to the pH of its surroundings.^44^ All these features established pMBA as the most commonly used Raman reporter in pH nanosensors,^45,46^ as well as a labeling molecule in SERS nanotags for bioimaging.^47^

Typically, all SERS experiments were carried out for self-assembled monolayers (SAMs) of pMBA grown overnight on CSLN substrates to ensure the formation of a defect-free and complete monolayer, unless the volume concentration of the thiol in solution was insufficient (as discussed further for the pMBA concentration-dependent SERS studies). In all cases, the substrates incorporate a dual metal Ag/Au layer, where the thickness of the former is a variable parameter and the latter is always 5 nm thick to provide protection from harsh chemicals. A typical SERS spectrum excited with a 532 nm laser observed for pMBA adsorbed from a 10^–4^ M ethanolic solution on CSLNs arrays is presented in Figure S8, while the vibrational assignment of the most intense SERS bands can be found in the inset of Figure S8.^43,45,48,49^ The appearance of vibration bands characteristic of carbonyl group stretching (around 1700 cm^–1^) and symmetric stretching of the carboxylate anion (around 1360 cm^–1^) indicates partial deprotonation of pMBA upon adsorption. The two bands that dominate the SERS spectrum of pMBA are due to benzene ring breathing modes: ν_12_ at 1075 cm^–1^ and ν_8a_ at 1585 cm^–1^.^22^ Their intensities are extremely useful to quantify the SERS signal of pMBA not only due to their large surface enhancement but also because of the independence of their intensity on environmental parameters (except those affecting molecular orientation). In the conducted evaluation of CSLNs’ performance, the main focus is on the 1585 cm^–1^ band as it is the strongest one in the SERS spectrum of pMBA.

**Figure 5:**
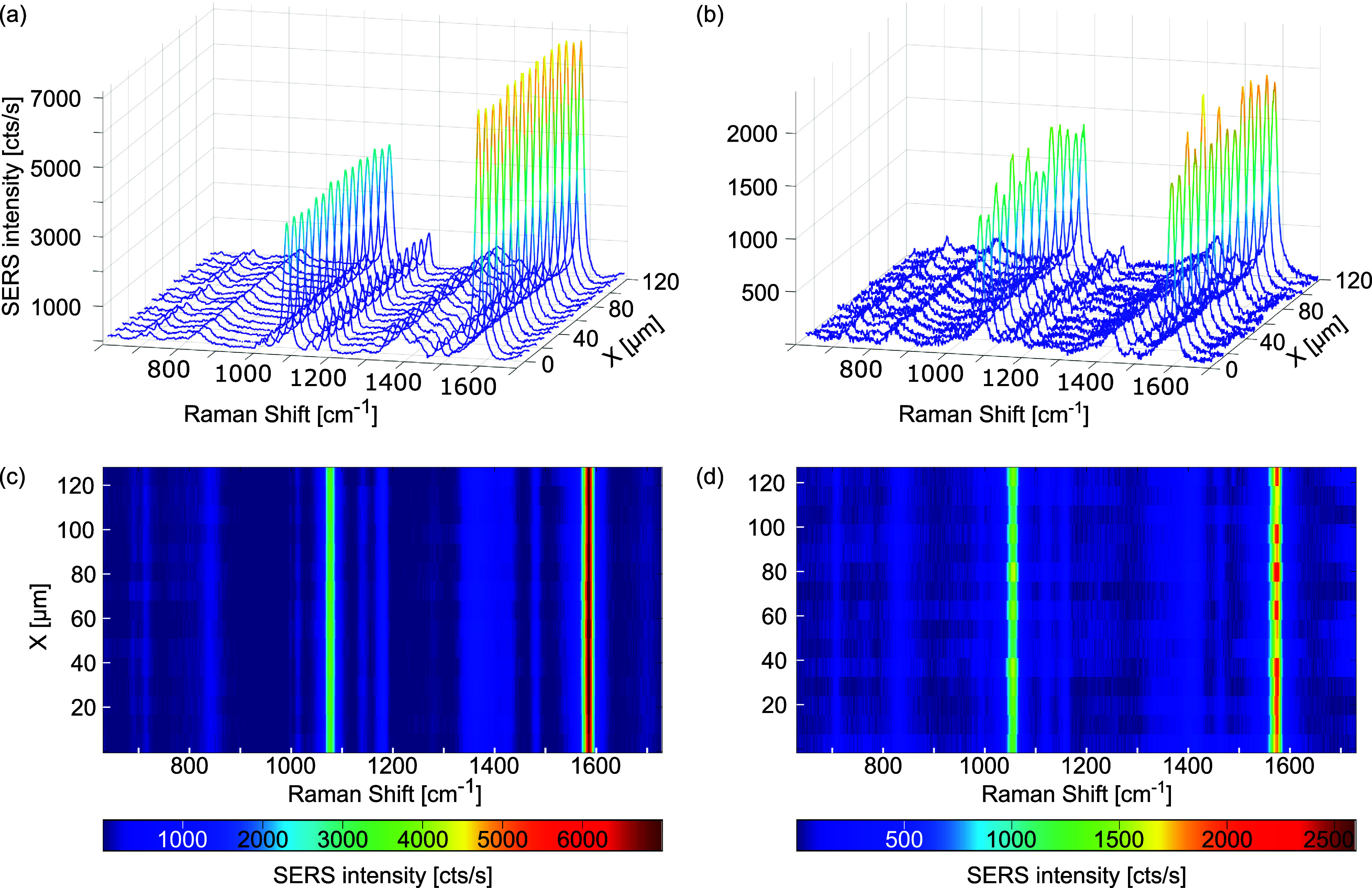
Data representative of excellent and mediocre SERS performance, illustrating the spectral fluctuations by three-axis graph visualization and corresponding waterfall plots of SERS spectra of 10^–4^ M pMBA collected using the mapping mode from 15 different spots (line profile) of the CSLN substrates fabricated with 0.2 wt % suspension of DNS, coated with 1.5 nm Ge, 40 nm Ag, and 5 nm Au for (a, c) 60 nm DNS and the 532 nm excitation line vs (b, d) 200 nm DNS and the 633 nm excitation line.

Prior to demonstrating a systematic procedure for optimizing the fabrication protocol of CSLNs as substrates for SERS spectroscopy, we present an explicit illustration of how the synthesis and measurement conditions can affect the SERS response of the substrates. Figure 5 compares the two sets of SERS spectra acquired for two CSLN substrates that differ in fabrication parameters and Raman data collection conditions, selected to illustrate excellent vs significantly worse quality in terms of their SERS performance. The top panel presents line profiles through the SERS map captured point by point and depicting the intensity distribution over the entire Raman shift range studied for pMBA adsorbed on CSLN substrates fabricated from 0.2 wt % DNS suspensions and coated with 1.5 nm Ge, 40 nm Ag, and 5 nm Au, but differing in DNS size and SERS spectrum excitation line: 60 and 532 nm (Figure 5a) vs 200 and 633 nm (Figure 5b), respectively. For the green laser and 60 nm DNSs, only minor fluctuations in the intensity of the SERS spectra can be observed, and the signal itself is very strong. For the red excitation line and 200 nm DNSs, one can see much more pronounced differences in the intensity of the SERS signal, which is also less enhanced, as shown in Figure 5b. The advantage of the waterfall plot presentation is that in addition to the differences in the intensity of the SERS spectra, one can better see the fluctuations in vibrational energy, which practically do not occur for 60 nm DNS and the 532 nm laser (Figure 5c), while their strong contribution for 200 nm DNS and the 633 nm laser (Figure 5d) suggests the inhomogeneous chemical state of the pMBA monolayer under measurement conditions. These results confirm the need to optimize the fabrication procedure of CSLNs as a key step for comprehensive SERS performance and to successfully meet all five criteria that define a high-quality SERS substrate.

**Figure 6:**
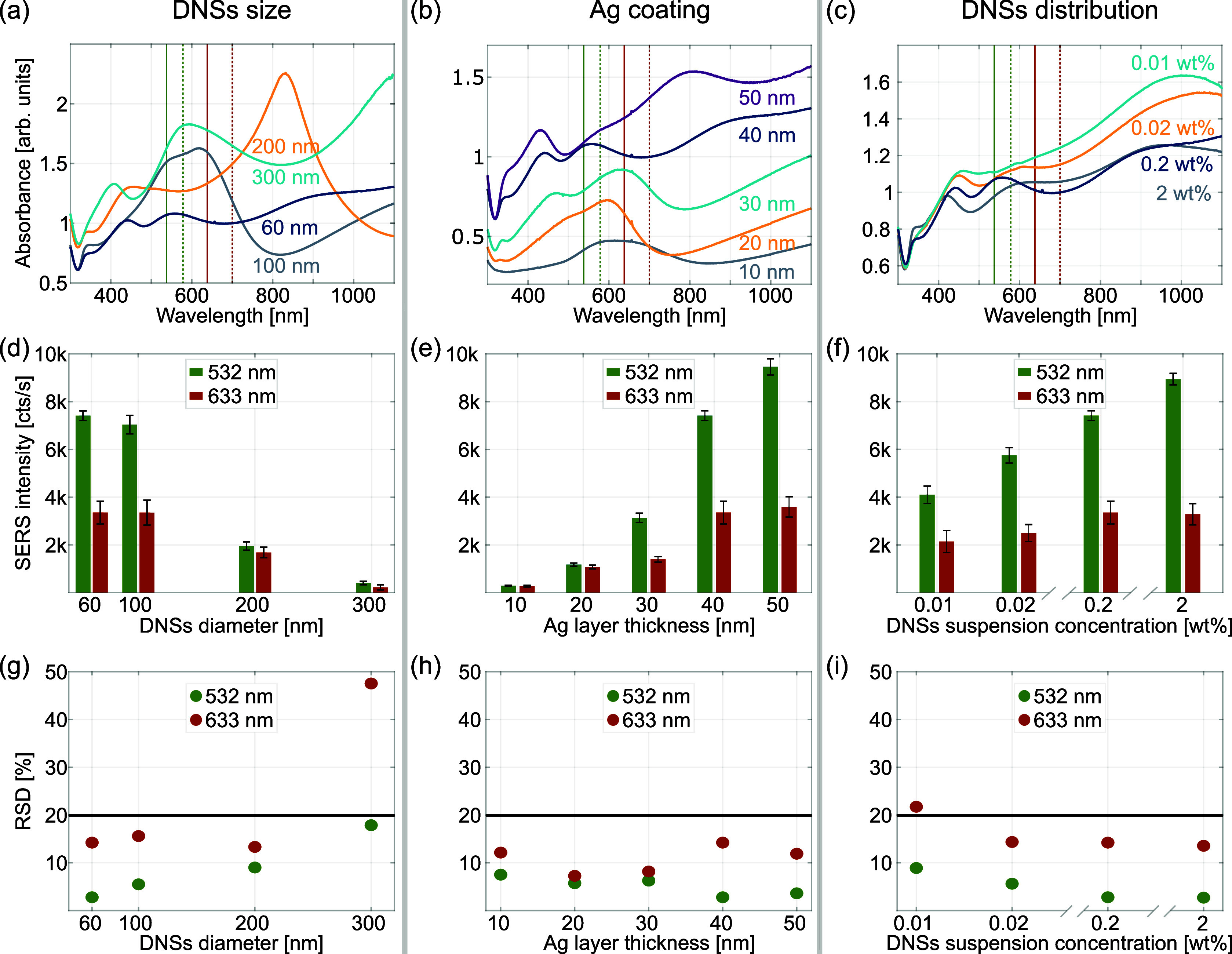
Absorbance curves analogous to the CSLN substrates analyzed in a top row of Figure 4, but with an additional 5 nm-thick gold layer on top presenting the effects of different: (a) diameter of DNS (fabrication parameters: 0.2 wt % suspension of 60–300 nm DNSs, metal multilayer: 1.5 nm Ge, 40 nm Ag, 5 nm Au); (b) silver layer thickness (0.2 wt % suspension of 60 nm DNSs, 1.5 nm Ge, 10–50 nm Ag, 5 nm Au); (c) mean distance between the DNSs (0.01–2 wt % suspension of 60 nm DNSs, 1.5 nm Ge, 40 nm Ag, 5 nm Au). The values of (a) the DNS diameter, (b) the nominal thickness of the silver layer, and (c) the concentration of DNS suspension are given in the legend. Effect of varying fabrication parameters of CSLNs analyzed in the top row on (d–f) SERS intensity of the pMBA band at 1585 cm^–1^ and (g–i) on the corresponding RSD values of SERS intensity. Each bar or point represents averaged data from 675 SERS spectra measured over an area of about 140 mm^2^. The solid black line at 20% indicates the maximum acceptable level of RSD in SERS analysis, as defined in Table 1. Green and red codes represent the results for a particular excitation wavelength (see the legend). The solid and dashed lines in a top row indicate the wavelengths corresponding to excitation lasers and Stokes-shifted analyzed SERS bands, respectively (see the text for details).

We begin by characterizing the impact of DNS size (Figure 6, left column), the thickness of the Ag layer deposited below 5 nm of Au (Figure 6, middle column), and the bulk concentration of DNSs in suspension (Figure 6, right column) on the SERS signal intensity of pMBA at 1585 cm^–1^. Absorbance spectra of the fabricated CSLNs are shown in Figure 6a–c. The average SERS signal and corresponding standard deviation (SD) acquired with two excitation wavelengths (532.0 and 632.8 nm) for the three analyzed fabrication parameters are shown in Figure 6d–f, while the RSD values of these results are plotted in Figure 6g–i. For the statistical needs of the SERS analysis, the signal was collected in each single case from a total of 675 various locations across three 60 × 60 μm^2^ areas on the substrate separated by at least 1 cm, each covering 225 distinct spots.

The dependence of SERS intensity of the 1585 cm^–1^ band with varying DNS sizes is similar for both excitation lines (see Figure 6d). The strongest signal is observed for diameters 60 and 100 nm, comparable for each laser, and in both cases, it decreases with a particle size. We recall here that the optimal conditions for SERS occur when both the incident and Raman scattered photons are plasmon-enhanced. For single resonance substrates, the plasmon peak should be located halfway between the wavelengths of the excitation laser and the Raman-shifted band of the analyte while covering them both.^50,51^ In the case of a multiply resonant substrate, it is preferred to match the energy of one plasmonic peak to the Rayleigh band and the other to the Stokes bands.^52,53^Figure 6a shows a red shift of the nanogap resonance with an increase in DNS diameter, which significantly changes the matching of this intense mode with the excitation wavelengths (the solid green/red vertical lines representing the 532 and 633 nm excitation lasers, respectively) and the corresponding absolute values of the Stokes-shifted band (position in maximum of the SERS band) of pMBA at 1585 cm^–1^ (dashed vertical lines at 581 and 703 nm). The strong SERS signal observed in Figure 6d for 60 and 100 nm nanospheres is a result of a very good overlap with the nanogap mode for both excitation lines, although the 633 nm laser is slightly red-detuned and thus provides a weaker SERS signal. This contrasts the significantly smaller SERS signal for the 200 and 300 nm DNS substrates whose nanogap modes are shifted to 830 and 1100 nm, respectively. In these two cases, there is a significant mismatch between the excitation/Stokes energies and the plasmon mode, resulting in weaker surface enhancement.

Interestingly, the decrease of the SERS intensity is much larger for the 532 nm laser: approximately 3.6- versus 2-fold for the 633 nm line when going from a diameter of 100 to 200 nm. Moreover, the observed magnitude of the SERS signal becomes nearly identical for these two excitation wavelengths. This trend cannot be fully explained by the simple effect of nanosphere size on the fraction of substrate area occupied by metal-coated DNS (see Figure S9 in Section S4 of the Supporting Information and discussion therein). As can be seen in the bar plot in Figure S9, surface coverage decreases gradually as the nominal diameter of the DNS increases, but the observed relationship does not perfectly reflect that in Figure 6d and fails to explain the effect of the excitation laser wavelength used.

To elucidate the latter, we recall that the second factor affecting the SERS intensity is the nanogap size and its impact on the overall EM field enhancement. Both the quadrupole and symmetric plasmonic modes are characterized by an enhanced electric field in the nanogap (see Figure 2c,d); however, the value of this enhancement decreases rapidly with an increasing gap size (Figure 3d). Thus, it is likely that this decrease is an additional, important reason for the sharp drop in SERS intensity for *d* > 100 nm. This is because the nanogap size increases with the diameter of the DNS for a constant metal thickness, as is illustrated by the SEM images in Figure S10 (the nanogap sizes are ca. 5, 20, 50, and 90 nm for the four analyzed DNS diameters). Based on the |*E*/*E*_0_|^4^ values calculated using FDTD results (Figure 2c,d), we estimate that the electric field enhancement is more than one order of magnitude larger for the nanogap/symmetric resonance than for the quadrupole mode. Thus, matching the excitation/Stokes band energy with the quadrupole mode yields weaker SERS enhancement when compared to the symmetric nanogap mode. These factors result in a similar SERS signal for both laser lines for the 200 nm DNS, because both the excitation lines and Stokes bands do not coincide with any plasmon resonance and exhibit only weak enhancement due to a gap of intermediate size (19 nm, according to Figure S10b). For *d* = 300 nm, the gap size becomes larger and the overall enhancement decreases even more. However, the overlap of the quadrupole mode by both excitation lines is comparable and rather mediocre, while there is a better match with the Stokes-shifted band for a green laser, which explains why the SERS signal excited with 532 nm wavelength is stronger than for the 633 nm. These results confirm how important the presence and size of nanogap are in the context of optimal SERS performance. For large nanogaps, regardless of the position of its plasmonic resonance with respect to the laser line Raman bands, the enhancement will not be satisfactory for visible-light excitation. An additional factor to consider is the surface density of DNSs and thus the number of the nanogaps acting as EM hot spots available to the molecules. A simple approximation confirmed by SEM images in Figure 4a (further supported by analysis of additional SEM data and the resulting surface coverage values presented in Figure S9) and surface density scaling of the random sequential adsorption indicates that for bigger DNS particles, their surface density and thus the number of nanogap SERS-active hot spots decrease, which also contributes to a general reduction of SERS intensity for larger DNS sizes.

Figure 6g presents the RSDs corresponding to the SERS intensities discussed above and plotted in Figure 6d. An exceptionally low RSD value of 2.5% is found for the 60 nm DNS excited using 532 nm, while for the larger 100 nm particles, it remains excellent at 5.5%. While RSD increases with nanosphere size, it does not exceed the required 20% limit (marked with a black solid line in Figure 6g and following ones in the same row) for the green laser excitation. Higher RSD values are noted for all DNS sizes in combination with the red laser; however, only for the *d* = 300 nm case do they exceed the 20% criterion (#1 in Table 1). The latter can be attributed to the fact that for diameters larger than 200 nm, the probability of DNS oligomerization increases drastically.

Two factors contribute to the rather general increase of RSD with the nanosphere diameter. First, for a constant metal thickness, it is most likely related to the increasing nanogap size, as a small nanogap is characterized by a very uniform distribution of the EM field. Thus, it is expected that the smallest gap observed for the 60 nm DNS (Figure S10a) exhibits simultaneously the smallest deviations and largest values of the SERS signal, confirming that ultrasmall nanogaps are essential for reproducible and strong SERS signal.^54^ A secondary reason is related to the variability of the size of metal-coated nanospheres and their distribution on the surface. The SD of the overall diameter increases for the larger DNS sizes (see the histograms of size distribution and resulting mean diameters and SD values in Figure S11 for the data determined from SEM images for the metal-evaporated nanospheres of various nominal diameters). Since the size of the nanospheres determines its resonance wavelength, a broader size distribution will increase the spread of the plasmonic peaks contributing jointly to the total optical response of a given area on the substrate and thus to the properties of the SERS signal. Indeed, the distribution of the size of CSLNs (see Figure S11 and relevant short discussion in Section S4 of the Supporting Information) nearly perfectly matches the trends observed for the RSD of the SERS signal in Figure 6g. Furthermore, the size of the nanosphere determines their surface number density, meaning that for a given laser spot size, the number of particles over which the SERS signal is averaged decreases with an increasing DNS diameter. As the arrays are random with a short-range correlation, the immediate vicinity of each particle is unique. Thus, in such an amorphous array even consisting of identical-sized particles, the individual resonances will vary due to different radiative couplings.^37,55^ Averaging the SERS signal over fewer nanospheres when using a constant laser spot size means that the variability of the smaller plasmon-active area of the substrate will be larger, further supporting the DNS-size-dependent RSD observations for both laser lines (Figure 6g).

Considering the factors discussed above, we ascribe the extraordinary spatial uniformity of the strong SERS signal observed for the smallest investigated DNS size and constant metal thickness mainly to the unique and statistically homogeneous geometry of CSLNs and their relatively dense packing. This combines the benefits of size-controlled core–shell-like nanoparticles with small nanogap structures and adequate averaging over a number of plasmonic features (see Figure 1e,f, together with Figures S1 and S10a) resulting in a well-defined optical response (Figures 4a and 6a).

Due to the very promising combination of both high surface enhancement and extremely low RSD values of the SERS signal for 60 nm DNSs, we proceed with this diameter and investigate the effect of Ag layer thickness on the SERS response of pMBA. Silver layers with thicknesses of 10, 20, 30, 40, and 50 nm along with 5 nm of gold on top were evaporated (absorbance spectra in Figure 6b, and the resulting SERS intensities and corresponding RSD values are shown in Figure 6e and Figure 6h, respectively). The behavior of the plasmonic peaks is similar to that of only the Ge and Ag coating (*cf*. Figure 4b and the related discussion), although the total metal thickness in this case is larger, as it also includes 5 nm of Au. For the same total amount of metal, only a slight resonance shift is noticeable, as depicted in Figure S7.

In Figure 6e, we observe a consistent behavior for both excitation wavelengths: the SERS intensity of the pMBA band at 1585 cm^–1^ gradually increases with a growing thickness of the Ag layer. The metal thickness increase is directly responsible for a decrease in the size of the nanogap, which is the dominant factor affecting the SERS signal intensity. For each substrate, except for the 50 nm Ag-coated one, a strong nanogap resonance overlapping the 532 nm (laser) and/or 581 nm (Stokes) wavelengths is observed (Figure 6b). However, the substrate with the thickest examined Ag layer (50 nm) exhibits a complicated absorbance curve resulting from the combined optical response of CSLN statistically containing fully or partially closed nanogaps for which none of the major resonances overlap with both laser lines or Stokes bands. For the 633 nm line laser and associated 703 nm Stokes band, the resonance maximum of the symmetric nanogap mode does not perfectly overlap any of these two wavelengths for the majority of examined thicknesses of the Ag layer; hence, the SERS signal is typically weaker than under green laser excitation. This detuning increases for thicker Ag layers, especially for 50 nm of Ag, but the significant decrease in the nanogap size compensates for the spectral mismatch, still providing decent SERS intensity.

The RSD dependence of the SERS signal on Ag thickness exhibits a pattern (Figure 6h) more complex than that in the case for the DNS size effect. For the 532 nm excitation line, we observe RSD values in the 5–10% range for the 10–30 nm Ag layer and even a lower one for 40 nm Ag with a very small, but well-controlled, ca. 5 nm gap (*cf*. Figure S10a). Further increase of the metal thickness by an additional 10 nm begins to close the nanogap in a stochastic way due to partial growth of nanoislands around the inside rim of the nanoaperture, which means that the size of the nanogap can no longer be fully controlled. The ultrasmall sub-5 nm gap still provides efficient hot spots for SERS but becomes inhomogeneous within the spot size. As plotted in Figure 3d, the EF is strongly variable on the minute differences in size within the sub-5 nm nanogap range. Thus, even a small variability of the gap yields very large changes of the EF. Therefore, when changing *h* of Ag from 40 to 50 nm, we observe a simultaneous increase in the RSD and SERS signals for the green laser line.

The pattern of the RSD dependence for both lasers is the same, except for the largest two values of Ag layer thickness, when the RSD is markedly different. The main cause behind this discrepancy is likely a different behavior of field enhancement at small gaps for the green and red lasers (*cf*. FDTD simulations in Figure 3d for the gap size ≤4 nm) and a smaller overlap of the red excitation line and the corresponding Stokes band with the plasmon peaks. The resulting weaker overall plasmon response of the whole nanostructure leads to a comparatively larger contribution from the local inhomogeneities of the metal coating, which create additional isolated, single-nm hot spots that ultimately increase the RSD of the SERS signal.

In general, the best conditions for large and uniform SERS enhancement are provided by operating near the gap closing, occurring in a uniform way around the circumference, while avoiding the formation of isolated sub-1 nm hot spots (see again the undesired in SERS experiment strong EF variations in Figure 3d for the gap size ≤4 nm). These conditions are best fulfilled for 60 nm DNS coated with 1.5 nm of Ge, 40 nm of Ag, and 5 nm of Au, showing RSD of the SERS signal of 2.5%. However, all considered metal thicknesses offer outstanding performance in terms of SERS signal homogeneity with RSD <7.5% for 532 nm laser and below 15% for 633 nm laser, thus easily within a tolerance range defined by criterion #1 in Table 1.

Subsequently, we optimized the surface density of the DNS by varying their suspension concentration when creating the templates for metal deposition. The results of this procedure, namely, SERS intensities of the pMBA band at 1585 cm^–1^ for varying bulk concentrations of DNS, are plotted in Figure 6f. The volume concentration of the DNSs determines the minimum center-to-center distance between particles deposited on the PDDA-coated glass support. With the increase of the DNS concentration, the number density of illuminated elements in the CSLN also increases, due to the effectively decreased mean interparticle distance between deposited DNSs (*cf*. SEM images in the bottom row of Figure 4c). The distance between the nanospheres determines the relative phase of the incident and scattered light impinging onto the nanostructures, and by changing this relation, all resonances are shifted spectrally, either to the blue or red—depending on the ratio between the resonance wavelength and the minimum center-to-center distance. The peak shifts are in the range of up to 10% of the single-particle response, thus playing a minor, though non-negligible, role, as the width of some of the plasmon peaks is rather broad. The more important parameter affecting SERS performance of the substrates is an increase of the particle number surface density with an increasing DNS concentration (see Figure S12 and the relevant short discussion in Section S4 of the Supporting Information). It leads to a spectral response collected from a larger number of illuminated (laser-activated) CSLNs, which simultaneously increases the SERS signal (Figure 6f) and, due to better averaging over a larger number of DNSs, decreases the RSD (Figure 6i). The reason for the decrease in the RSD values is similar to that observed for a decreasing size of the DNS particle (Figure 6d,g). The concentration-dependent RSD values are markedly smaller for green laser excitation (below 10%) than for the red one (13–22%). This excitation laser wavelength dependence is also present for the substrates with a Ag layer (and 5 nm of Au) thicker than 30 nm, as visible in Figure 6h. As discussed previously, this difference is caused already by the geometry of the gap of an individual CSLN; thus, a decreasing particle density is unlikely to significantly alter the RSD ratio between the green and red lasers. The lowest and nearly identical RSD values of 2.7 and 2.8% occur for the concentrations of 2.0 and 0.2 wt %, respectively (Figure 6i). Note that if we analyze 225 SERS spectra from an area of about 3600 μm^2^ for the highest studied concentration of DNS, then the RSD reaches an extremely low value of 1.9%. Despite the slightly better SERS signal for the 2.0 wt % concentration, for further evaluation, we select the CSLN substrates fabricated with 0.2 wt % suspension due to a significantly smaller amount of material used in their preparation, making it a more cost-effective option with respect to the end cost of the substrate fabrication. Guided by the preceding analysis, all further characterization of SERS performance utilizes CSLN substrates composed of 60 nm DNSs deposited from a suspension of 0.2 wt % and coated sequentially with 1.5 nm Ge, 40 nm Ag, and 5 nm Au.

The next considered quality of the CSLNs is the EF. It is a quantitative parameter that allows comparison of various substrates in terms of their SERS performance. Unfortunately, methods used for calculating the EF are often highly questionable. Common problems include neglecting the resonance Raman contribution to the overall EF and using unjustified, rough estimations of experimental parameters, with molecular surface coverage being the most difficult one to determine accurately.^56^ Indeed, recent works have highlighted the lack of standard procedures in determining EF values, in terms of both measurement conditions and analyte selection.^21,57,58^

In order to minimize all these pitfalls, we use pMBA and pyridine (Pyr) to evaluate the SERS activity of the CSLN substrates, mainly because these molecules do not exhibit resonance Raman cross sections under experimental conditions. These analytes were also chosen because they are among the most recognized adsorbates by the SERS community—pMBA as a routine Raman reporter for pH sensing, and Pyr as the first molecule for which the SERS effect was experimentally observed.^59^ Moreover, we adopt an approach in which the EF is determined with reference to a standard electrochemically roughened silver substrate produced by an oxidation–reduction cycling (ORC) procedure. An additional benefit is that the presented results involve SERS measurements carried out using identical experimental parameters (including exactly the same ORC roughening procedure and Raman setup) as the reference data from Ambroziak et al.^60^ In this work, the EF for 0.05 M Pyr in 0.1 M KCl adsorbed on a reference ORC-roughened silver electrode was found to be equal to 4.6 × 10^5^, as determined for the Pyr SERS breathing mode at 1004 cm^–1^ (ν_1_, Wilson notation).^60,61^ Here, applied calculations involve comparing the intensity of the SERS signal acquired under specific measurement conditions to that obtained under identical conditions for Ag ORCs with known surface enhancement parameters and determining the EF for CSLN substrates on that basis. This procedure provides EF values for CSLNs up to 1.5 × 10^6^ when using only Ge and Ag, and 0.62 × 10^6^ with an additional 5 nm Au layer. Such estimated EF values for Pyr for both excitation lines are summarized in Table 2. These parameters, determined from measurements over an area twice larger than the minimum recommended size, indicate excellent sensitivity of CSLN substrates for SERS spectroscopy (criterion #4 in Table 1). Moreover, these experimental results are in reasonable agreement with the theoretical EF values predicted for the nanogap mode (see Figure 3d), which are on the order of 0.3 × 10^6^ for the green laser when the gap size is just below 5 nm (as determined from the SEM image in Figure S10a). This confirms the dominating role of the nanogap plasmon mode in the SERS response of the CSLNs.

**Table 2:** Determined Values of an Enhancement Factor (EF) for Pyr and SERS Gain (*G*_SERS_) for pMBA Adsorbed on CSLNs Fabricated with 0.2 wt % 60 nm DNSs, 1.5 nm Ge, and 40 nm Ag with and without 5 nm Au

**analyte; concentration and SERS band**	**parameter**	**40 nm Ag**	**40 nm Ag, 5 nm Au**
*532 nm laser*			
Pyr; 5 × 10^–2^ M in 0.1 M KCl at 1010 cm^–1^	EF	1.5 × 10^6^	0.62 × 10^6^
pMBA; 1 × 10^–4^ M, at 1585 cm^–1^	*G*_SERS_	177	113
*633 nm laser*			
Pyr; 5 × 10^–2^ M in 0.1 M KCl at 1010 cm^–1^	EF	0.46 × 10^5^	0.33 × 10^6^
pMBA; 1 × 10^–4^ M, at 1585 cm^–1^	*G*_SERS_	1486	150

The EF for pMBA cannot be estimated using the above method due to a lack of appropriate reference data. Therefore, the so-called SERS gain (*G*_SERS_) is used instead for this analyte (see Table 2 for the determined values). It is calculated as a ratio of the intensity of the SERS signal for the CSLN substrates and the Raman signal for the bare and 5 nm Au-coated 40 nm planar silver layers (normalized to laser power and acquisition time). This approach allows for directly establishing the impact of the CSLN geometry, with material composition aside from SERS performance. *G*_SERS_ is a less common indicator of SERS substrate sensitivity than EF, but it enables a straightforward evaluation of the signal amplification by a given nanostructure relative to a planar metallic film^62^ or a normal Raman signal for a volume sample of the same concentration.^63^ Advantageously, it is not affected by uncertainty related to the estimation of the number of molecules probed in SERS and the reference measurement. The SERS signal for CSLNs excited with 532 nm laser for the 1585 cm^–1^ band of pMBA is more than two orders of magnitude higher than for the reference planar layers, achieving *G*_SERS_ of 177 for 40 nm Ag and 113 when using the additional 5 nm of Au. This difference is even more pronounced for the red laser, for which *G*_SERS_ is an order of magnitude larger for the substrate without 5 nm Au than in its presence, among others as a result of an almost four times stronger SERS signal for CSLNs for the former. While these values are valid for the CSLN substrates considered optimal in terms of their comprehensive performance, it is apparent that *G*_SERS_ for pMBA is much smaller than the actual EF value. This is due to the significantly smaller number of molecules experiencing the enhanced EM field of the hot spots in the nanogaps relative to the reference planar substrates measurement.

**Figure 7:**
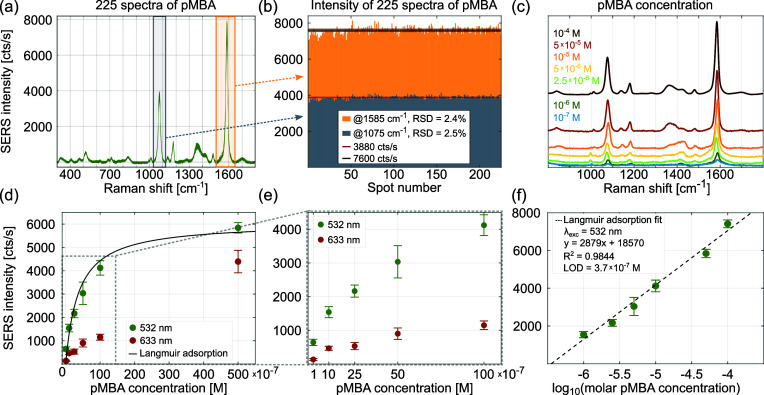
(a) SERS spectra of 1 × 10^–4^ M pMBA adsorbed on an amorphous array of CSLNs, collected from 225 substrate spots covering an area of around 3600 μm^2^. (b) Intensity variation of two characteristic pMBA bands obtained from the data in (a) showing an RSD value of ≤2.5%. (c) SERS spectra excited with a 532 nm laser for varying molar concentrations of pMBA from 10^–7^ to 10^–4^ M. (d) SERS intensity of the 1585 cm^–1^ band vs pMBA molar concentration for the two excitation lines and the fitted Langmuir adsorption model for the green laser and (e) its zoomed-in region up to 10^–5^ M. (f) Linear relationship between the SERS intensity of the pMBA band and the logarithm of the analyte concentration (parameters of the fit and LOD for pMBA given in the legend). All data were acquired for optimized CSLNs, fabricated with 0.2 wt % suspension of 60 nm DNS, coated with 1.5 nm Ge, 40 nm Ag, and 5 nm Au. SERS data in (a–c) and (f) were acquired with a 532 nm laser.

In Figure 7a, one can see the excellent repeatability of the 225 SERS spectra of pMBA collected from a square-shaped area of about 3600 μm^2^ (excited with a 532 nm laser). This is illustrated further in Figure 7b with a bar plot of SERS intensities for said spectra acquired from 225 different substrate spots for pMBA. The obtained RSD values are 2.4 and 2.5% for bands at 1075 and 1585 cm^–1^, respectively. Increasing the analyzed substrate area to 140 mm^2^ and the number of SERS spectra to 675 nm only slightly increases the RSD value to 2.8%, far exceeding the requirements for a high-quality SERS substrate (criterion #1 in Table 1). Maps illustrating the spatial distribution of the uniformity of SERS signal intensity of the strongest pMBA band, as well as the small spread of deviations from the average spectrum for each of the three areas studied (225 spectra each), clearly confirm the excellent spatial uniformity of the CLSN substrate with optimized geometry (see Figure S13 and brief discussion in Section S4 of the Supporting Information).

This extraordinarily low RSD value of 2.8% obtained from a large substrate area (>100 mm^2^) and a large number of points (nearly 700) is a cutting-edge result when considering the spatial uniformity of SERS-active substrates. One of the few examples of such exceptionally low RSD of the SERS signal for a comparable number of points are the very recently reported substrates based on ultrastrong coupling between the LSPR of gold nanoparticles and Fabry-Pérot nanocavities, which provide RSD of 3% for 625 locations within a 30 × 30 μm^2^ area.^64^ Similar RSD values of less than 3%, although for an undefined number of SERS measurements, were also demonstrated for substrates with nanogaps based on a different geometry than proposed here; however, the structures were produced with sophisticated deep UV lithography.^65^

The performance of optimized CSLNs in the SERS-based quantitative analysis of pMBA is used to further characterize the fabricated substrate. In Figure 7c, we plot SERS spectra obtained for pMBA concentration values ranging from 10^–7^ to 10^–4^ M excited with the 532 nm wavelength. The changes of the SERS intensity with a varying pMBA concentration are analyzed using the signal of the 1585 cm^–1^ band for the two examined laser lines. As seen in Figure 7d and the magnification of low concentration range in Figure 7e, the SERS signal initially increases rapidly with rising pMBA concentration and reaches a quasi-plateau when the concentration is ≥10^–5^ M. This saturation of the SERS signal is typical of surface adsorption^66^ and is consistent with the Langmuir adsorption isotherm model (see the fit in Figure 7d for the green laser), for which the surface coverage of the analyte is given by
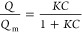
1where *Q*/*Q*_m_ is the surface coverage given by a ratio of the SERS intensity of the analyzed band for a particular concentration of pMBA molecules in solution (*C*) and when all adsorption sites are occupied, while *K* is the adsorption equilibrium constant. The observed trend suggests that for pMBA concentrations lower than 10^–5^ M, thiol-metal interactions are partially hindered, with only a portion of the active sites being occupied by pMBA molecules. A probable cause of this behavior is the progressive dissociation of the carboxyl groups, which is supported by the presence of the symmetric stretching vibration band of COO^–^ groups in the SERS spectra (see Figure 7a and the band assignment in Figure S8), thus evidencing a partial deprotonation of pMBA already for the 10^–4^ M concentration. The carboxylate form of pMBA may preferentially interact with the metal and/or electrostatically repel molecules diffusing out of solution, both of which will hinder adsorption and lower the resulting surface concentration of the thiol (even reaching a submonolayer regime). Next, the concentration-dependent SERS data for the 532 nm excitation line and the 1585 cm^–1^ band are used to establish the limit of detection (LOD) of pMBA. A linear fit to the semilogarithmic plot of the SERS-signal-vs-pMBA-concentration data is performed and is plotted in Figure 7f. Using a protocol involving the analysis of a blank sample (see Materials and Methods for details), we obtain the LOD as 3.7 × 10^–7^ M. It is worth highlighting that the LOD for pMBA obtained with the optimized CSLNs is one order of magnitude lower (and thus better) than that for commercial gold SERS substrates, while the RSD is also reduced by one order of magnitude.^67^ This demonstrates the improved sensitivity and precision of the SERS measurement with the substrates developed in this work.

The shelf life of an SERS substrate is also a critical parameter for real-world applications and mass production. Therefore, we compare the SERS signal of optimized CSLN substrates soon after fabrication and after 4 months of storage before functionalization with 10^–4^ M of pMBA. This analysis is performed for substrates with and without an additional 5 nm Au layer and the results are presented in Figure S14a and Figure S14b, respectively. While changes of the SERS signal were not monitored weekly, the 4-month storage of the substrates significantly exceeds the recommended period of 1 month for testing temporal stability (criterion #3 in Table 1). The drop of the SERS signal of pMBA at the 1585 cm^–1^ band is 18.7% for the CSLNs covered with a Ge/Ag/Au multilayer and 3.6% for the Ge/Ag one. For the 1075 cm^–1^ band, the decrease of SERS intensity does not depend significantly on the presence of the Au layer and is equal to 13.6% with the Au and 15.8% without it. The smaller decrease in intensity for the pMBA SERS signal at 1585 cm^–1^ in the absence of Au is surprising. However, due to the increased complexity of the multilayered system, it is possible for material migration between layers to occur,^68^ which would result in a modification of plasmonic characteristics and thus the observed change in SERS intensity. A second possibility is a subtle change of the orientation of the pMBA molecular monolayer due to surface restructuring by segregation within the metallic layer, the former of which can result in a change in the relative intensity ratio of the SERS bands.^66^

The previously made decision to apply a 5 nm Au film coating is further justified by the significantly lower and competitive RSD values of the SERS signal compared to using Ag alone. The lower RSD value with gold capping is confirmed for both freshly prepared CSLN substrates (for a 532 nm excitation and the 1585 cm^–1^ band, the RSD is 2.8% with Au vs 11.3% without) and 4 months after fabrication (6.2 vs 8.5%, respectively). Additionally, Au-coated substrates are substantially more resistant to aggressive reagents; for example, they remain stable when exposed to chloride ions in solution.

Substrate-to–substrate reproducibility of CSLNs was also verified by collecting SERS response of pMBA for six substrates fabricated over a 5-month time frame. The evaluation is based on SERS measurements from a total of more than 20 locations on these six substrates (see Figure S15), further cut into smaller pieces. Each spot corresponds to 225 spectra from a 60 × 60 μm squared area. The variability of the SERS signal on this sample of more than 4500 spectra from different fabrication batches is more than satisfactory, as evidenced by an RSD of 14.7%. We therefore consider that CSLNs meet the condition for high-quality SERS substrates (criterion #2 in Table 1).

In the final step of the evaluation of CSLN SERS performance, we use Pyr and methyl orange (MO) under off-resonance conditions to examine SERS capability toward the detection of analytes under different charge states, i.e., neutrally/positively/negatively charged adsorbates. Specifically, Pyr can undergo partial protonation in aqueous solution even at neutral pH, while MO is a halochromic anionic/neutral azo dye that contains a pH-sensitive chromophore. As can be seen in Figure S16, a reliable and consistent SERS signal (*cf*. the RSD values of the SERS intensity given in the legend) is readily obtained for these two analytes next to pMBA for the optimized CSLN substrates, demonstrating the versatility of this platform (criterion #5 in Table 1).

## Conclusions

Nanogap plasmonic structures garner considerable attention in various fields of science and engineering due to large EM field enhancement and subwavelength mode volumes of excited surface plasmon resonances. However, their real-world applicability is limited by the lack of convenient and cost-effective nanofabrication methods capable of spatial control of matter in the sub-10 nm regime on macroscopic-size substrates. To address this issue, we presented a simple and clean-room-free procedure for fabricating plasmonic substrates of a few cm^2^ in size, featuring nanogaps of engineered dimensions, including sub-10 nm ones. The multiresonance optical response of proposed CSLNs is susceptible to precise tuning by a simple adjustment of one or more of over 10 available fabrication parameters. Notably, already by altering the evaporated metal layer thickness, nanoparticle diameter, interparticle spacing, and material selection, the characteristic plasmonic resonances can be placed virtually anywhere within the UV–vis-NIR spectral range. The dominant spectral resonance is associated with the symmetric nanogap mode which concentrates light in deep subwavelength volumes, yielding EFs in SERS spectroscopy on the order of 10^6^ in practice, but potentially able to reach EF of 10,^9^ in line with the theoretically predicted values for subnm nanogaps.

In molecular detection experiments using pMBA and supplementary analytes, we demonstrated that the proposed nanoarchitecture is suitable for comprehensive SERS analysis. We addressed issues related to characterization methodologies, providing insights into the suitability of this architecture for SERS spectroscopy applications, and showed the versatility and robustness of the platform for the purposes of this method. Specifically, it was demonstrated that the CSLN substrates fulfill all five criteria defining a high-quality SERS substrate:^22,23^ uniform signal intensity with variability as low as 2.8%, decent EF of ∼10^6^, sufficient substrate-to-substrate reproducibility (signal variation <15%), high temporal signal stability (signal loss <4%), and documented SERS activity toward three analytes with different charge states. The Langmuir adsorption model was also confirmed for pMBA, and the ability to quantitatively detect this analyte using SERS with a LOD of 10^–7^ M was demonstrated. A thorough investigation of the influence of the architecture parameters of CSLN substrates on the optical response and corresponding SERS signal revealed the capabilities of the platform in real-life applications, proving that the established methodology enables easy, cost-effective, and macroscopic-scale fabrication of nanostructured plasmonic substrates with single-nm features. This combination of typically mutually exclusive attributes makes the proposed nanostructures outstanding candidates for other applications requiring a multimodal plasmonic response, high-precision tuning capabilities, and excellent reproducibility.

## Materials and Methods

### Substrate Preparation

Microscopic glass slides (EquiMed) cut into 2.5 cm × 2.5 cm pieces were thoroughly rinsed with ethanol (Sigma-Aldrich, HPLC, ≥99.8% pure) and pure water (0.12 μS/cm) and dried with nitrogen gas (N5.0 purity). Then, they were cleaned in oxygen plasma using the Diener Zepto RIE set to 30% of power for 5 min. These two steps were repeated twice.

An aqueous solution of poly(diallyldimethylammonium chloride) (PDDA polymer, Merck, 99.5% pure) at a concentration of 0.2 wt % was then pipetted onto the glass and rinsed after 40 s. The same protocol was repeated using an aqueous suspension of sulfate latex beads (composed of sulfate-functionalized polystyrene) of the desired diameter and appropriate concentration (Thermo Fisher Scientific, 8% w/v, diameters: 0.3, 0.2, 0.1, and 0.06 μm). Finally, various types of coatings were evaporated onto the substrates using a PVD75 ePVD system (Kurt J. Lesker) with evaporation rates ranging from 0.3 to 1 Å/s. Silver and gold pellets (99.99% pure) as well as germanium pieces (99.999%) were purchased from Kurt J. Lesker. All the processing gases used for plasma cleaning and evaporation, i.e., O_2_ and N_2_, were of N5.0 purity. Substrates are always placed within a specific area around the center of the plate used to mount the samples in the deposition system that was experimentally confirmed to preserve a perpendicular deposition angle, which ensures symmetrical CSLN growth. This step of the fabrication protocol is key for precisely controlling the uniformity of multiple samples within one process as well as to guarantee process-to-process reproducibility.

### Substrate Characterization

UV–vis spectra of the plasmonic substrates were collected in a transmittance mode using a Metash UV-6100 UV–vis spectrophotometer and/or a Woollam RC2 ellipsometer (D+NIR model) operating in the wavelength range of 190–1100 and 193–1690 nm, respectively. SEM images were taken using a Zeiss Sigma scanning electron microscope using the InLens detector. The accelerating voltage varied from 8 to 14 kV, depending on the sample and measurement angle.

Nanogap and CSLNs sizes, along with their SDs and resulting surface coverage for metal-coated DNS diameter, were determined using ImageJ software (Figures S9–S12). For each SEM image, the contrast was adjusted to most accurately represent the actual size of the nanospheres. The area of each circle was calculated with ImageJ and converted to the diameter if needed. The SD was subsequently calculated by using Microsoft Excel software.

### FDTD Calculations

Finite-difference time-domain (FDTD) simulations were performed with Ansys Lumerical 3D FDTD commercial software. Optical properties of an amorphous array of CSLNs were simulated with the use of periodic boundary conditions and a subwavelength size of the unit cell equal to 150 nm, which corresponds to the average distance between particle centers observed in the experiment. Since the structure exhibits subnanometer details, simulations were carried out with 0.5 nm resolution in the whole simulation region, while in the nanogap, additional mesh with a size of 0.3 nm was used. Complex refractive indices of metals used in the simulations, namely, Ag, Au, and Ge, were taken from ellipsometric measurements carried out in the experiment with use of a dual rotating compensator spectroscopic ellipsometer (Woollam RC2). Dielectric spheres (with a refractive index of 1.59 to model latex) were placed on a SiO_2_ glass substrate with a refractive index taken from Palik.^42^ The structure was illuminated from the top (air) side by a broadband (300–1100 nm) plane wave source; transmitted (and reflected) power was collected with a monitor placed below the structure within the substrate. The incident light was polarized in the plane of incidence. Prior to the main simulations, convergence testing of results was performed with use of multiple parameters including simulations time, resolution, and number of perfectly matched layers*.*

### Chemicals for the Molecular Layer Formation

*p*-Mercaptobenzoic acid was purchased from Sigma-Aldrich (Merck), while pyridine and methyl orange were supplied by Ubichem Limited and Thermo Scientific, respectively. KCl and absolute ethanol were purchased from POCh S.A. All chemicals were used as received without any purification. The aqueous solutions were prepared using ultrapure water with resistivity controlled at 18.2 MΩ·cm, obtained with a RephiLe Genie Water system (RephiLe Bioscience, Ltd.).

### Raman Instrumentation

SERS measurements were performed using a LabRam HR800 (HORIBA Jobin Yvon) Raman spectrometer, coupled to a BX41 Olympus confocal microscope. All spectra were collected in a backscattering configuration. A diode-pumped, frequency-doubled Nd:YAG laser operating at a wavelength of 532.0 nm was used as an excitation source. Alternatively, a built-in He–Ne laser providing a 632.8 nm line was used. The output power on the sample was below 3 mW for both applied laser lines, focused with a 100× objective lens (Olympus, NA = 0.9) or 50× long-distance objective (Olympus, NA = 0.5). The scattering signal after passing through an edge filter was dispersed using a holographic grating with 600 grooves/mm onto a Peltier-cooled, charge-coupled device (CCD) detector (1024 × 256 pixel) with a working temperature of −70 °C. The system was calibrated using the Raman band at 520 cm^–1^ of a silicon wafer, which was also used to control the temporal stability of the Raman setup performance.

### Sample Preparation and Data Collection in SERS Spectroscopy

SERS activity of the fabricated nanostructures was examined with pMBA as a probe molecule. Functionalization of the surface was carried out by submerging the entire, freshly prepared substrate in pMBA solution in ethanol overnight before SERS studies for each of the studied CSLNs, including batch-to-batch comparison. Washing the chemically modified nanostructures with copious amounts of ethanol and drying in air were applied prior to SERS measurements. To evaluate the stability of the SERS performance, the as-prepared substrates were first stored in a desiccator for around 4 months and next functionalized with pMBA, according to the procedure described below.

A 10^–4^ M pMBA solution was used as a typical concentration for testing the SERS response of the nanostructures prepared under various conditions of synthesis. Further dilution with ethanol to 10^–7^ M was used to identify the LOD of pMBA by the SERS method for the examined substrates.

SERS spectra of pMBA were typically collected with a 100× lens for each point of the 15 × 15 grid within a previously defined rectangular range of 61 μm × 58 μm using an automated microscope stage. Three various regions were analyzed for each substrate and particular excitation wavelength with around a 1.0 cm lateral spacing from each other. This means that 225 SERS spectra were analyzed for each area, while the SERS signal was measured at 675 various spots for a particular substrate, separated by a macroscopic distance between the three examined areas. Identical conditions were applied for SERS measurements of 10^–4^ M of MO adsorbed from aqueous solution.

The SERS measurements of pyridine, used to evaluate the EF, were collected using the same instrument and method as those described for pMBA, with two differences. First, a ×50 lens was used instead of a ×100 lens. Second, rather than using overnight adsorption, the sample was illuminated with a laser through a drop of solution. An appropriate volume of 0.05 M pyridine aqueous solution in 0.1 M KCl was pipetted onto the substrates placed on a glass slide, allowing the solution to flow slightly from the sample onto the slide. The purpose of this approach was to obtain a flatter layer of pyridine solution on the substrate to avoid light lensing from a spherical droplet. The whole procedure was aimed at reproducing the measurement protocol used in the work by Ambroziak et al.^60^

The acquisition time for a single accumulation varied from 0.5 to 3 s, while the number of accumulations was 2 for each spectrum. Single-point SERS spectra were acquired typically in ranges of 200–1800 and 600 to 1750 cm^–1^ for the 532.0 and 632.8 nm excitation lines, respectively.

### SERS Data Analysis

The accumulation time varied between the experimental series, but all the values of SERS intensity were recalculated to cts s^–1^ (counts per second) to allow their direct comparison. SERS spectra were first baseline corrected in LabSpec 5 software prior to further analysis. VBA macro executed in Microsoft Excel facilitated the determination of the average (arithmetic mean) maximum intensities of the selected pMBA and MO bands and their relative standard deviation (RSD) within the examined SERS spectra set. RSDs were defined as the SD divided by the average SERS intensity in the maximum of a given band and expressed in percentage and used to evaluate the spatial reproducibility of the SERS signal for a particular CSLN substrate. Three-axis graph visualization and waterfall graphs of line profiles of SERS spectra were plotted using LabSpec 5 software. 2D visualizations of SERS maps for pMBA were plotted with MATLAB software. For the correlation map plot, the mean spectrum was calculated for each map, and the maximum value of the 1585 cm^–1^ band was extracted. Then, the maximum value of the same band from each spectrum was divided by this mean value.

Fitting of the SERS spectra obtained for pyridine was performed using a house-made Python Scripts suite that has already proven itself in similar applications.^69,70^ The LOD for pMBA was determined by using a blank sample corresponding to the SERS intensity for the substrate without adsorbed pMBA (denoted as *I*_0_). A plot of *I*_SERS_ vs log_10_ from the pMBA concentration was generated, with the line *I*_*S*ERS_ = *I*_0_ parallel to the abscissa. The intersection of this line with a linear fit was projected onto the abscissa, yielding log_10_(LOD). The LOD was then determined as 10 raised to this power.

## Supporting Information Available

Details of the CSLN fabrication procedure and visualization of substrate homogeneity at the nanoscale (SEM images) and macroscale (photography); SEM micrographs showing structural details of CSLN geometry; comparison of the presented fabrication protocol with state-of-the-art methods; SEM and absorbance results illustrating the effect of the Ge wetting layer; FDTD simulations of optical properties for regular CS nanostructures (with fixed geometry or variable metal layer thickness); analysis of the effect of key simulation parameters on the nanogap resonance’s position and peak intensity; experimental results of the effect of Au thin film addition on absorbance; typical SERS spectrum of pMBA with vibrational assignment of the bands; size- and concentration-dependent surface coverage of the substrate (as determined from SEM imaging); determined sizes of the nanogap for CSLNs with different geometries (analysis of SEM images); histograms of dispersion of diameter size for metal-coated DNSs (based on SEM images); SERS results confirming: spatial homogeneity, sufficient temporal stability; high substrate-to-substrate reproducibility; and SERS activity toward three different analytes (PDF)

## References

[ref1] Baumberg J. J.; Aizpurua J.; Mikkelsen M. H.; Smith D. R. Extreme nanophotonics from ultrathin metallic gaps. Nat. Mater. 2019, 18 (7), 668–678. 10.1038/s41563-019-0290-y. 30936482

[ref2] Boroviks S.; Lin Z. H.; Zenin V. A.; Ziegler M.; Dellith A.; Gonçalves P. A. D.; Wolff C.; Bozhevolnyi S. I.; Huang J. S.; Mortensen N. A. Extremely confined gap plasmon modes: when nonlocality matters. Nat. Commun. 2022, 13 (1), 3105 10.1038/s41467-022-30737-2. 35661728 PMC9166740

[ref3] Yoo D.; de León-Pérez F.; Pelton M.; Lee I. H.; Mohr D. A.; Raschke M. B.; Caldwell J. D.; Martín-Moreno L.; Oh S. H. Ultrastrong plasmon-phonon coupling via epsilon-near-zero nanocavities. Nat. Photonics 2021, 15 (2), 125–130. 10.1038/s41566-020-00731-5.

[ref4] Zhu W. Q.; Esteban R.; Borisov A. G.; Baumberg J. J.; Nordlander P.; Lezec H. J.; Aizpurua J.; Crozier K. B. Quantum mechanical effects in plasmonic structures with subnanometre gaps. Nat. Commun. 2016, 7, 11495 10.1038/ncomms11495. 27255556 PMC4895716

[ref5] Cui X. M.; Qin F.; Lai Y. H.; Wang H.; Shao L.; Chen H. J.; Wang J. F.; Lin H. Q. Molecular Tunnel Junction-Controlled High-Order Charge Transfer Plasmon and Fano Resonances. ACS Nano 2018, 12 (12), 12541–12550. 10.1021/acsnano.8b07066. 30462918

[ref6] Altug H.; Oh S. H.; Maier S. A.; Homola J. Advances and applications of nanophotonic biosensors. Nature Nanotechnol. 2022, 17 (1), 5–16. 10.1038/s41565-021-01045-5. 35046571

[ref7] Cai H.; Meng Q.; Zhao H.; Li M.; Dai Y.; Lin Y.; Ding H.; Pan N.; Tian Y.; Luo Y.; et al. High-Throughput Fabrication of Ultradense Annular Nanogap Arrays for Plasmon-Enhanced Spectroscopy. ACS Appl. Mater. Interfaces 2018, 10 (23), 20189–20195. 10.1021/acsami.8b04810. 29799180

[ref8] Jin H. M.; Kim J. Y.; Heo M.; Jeong S. J.; Kim B. H.; Cha S. K.; Han K. H.; Kim J. H.; Yang G. G.; Shin J.; et al. Ultralarge Area Sub-10 nm Plasmonic Nanogap Array by Block Copolymer Self-Assembly for Reliable High-Sensitivity SERS. ACS Appl. Mater. Interfaces 2018, 10 (51), 44660–44667. 10.1021/acsami.8b17325. 30480431

[ref9] Langer J.; Jimenez de Aberasturi D.; Aizpurua J.; Alvarez-Puebla R. A.; Auguié B.; Baumberg J. J.; Bazan G. C.; Bell S. E. J.; Boisen A.; Brolo A. G.; Choo J.; Cialla-May D.; Deckert V.; Fabris L.; Faulds K.; García de Abajo F. J.; Goodacre R.; Graham D.; Haes A. J.; Haynes C. L.; Huck C.; Itoh T.; Käll M.; Kneipp J.; Kotov N. A.; Kuang H.; Le Ru E. C.; Lee H. K.; Li J. F.; Ling X. Y.; Maier S. A.; Mayerhöfer T.; Moskovits M.; Murakoshi K.; Nam J. M.; Nie S.; Ozaki Y.; Pastoriza-Santos I.; Perez-Juste J.; Popp J.; Pucci A.; Reich S.; Ren B.; Schatz G. C.; Shegai T.; Schlücker S.; Tay L. L.; Thomas K. G.; Tian Z. Q.; Van Duyne R. P.; Vo-Dinh T.; Wang Y.; Willets K. A.; Xu C.; Xu H.; Xu Y.; Yamamoto Y. S.; Zhao B.; Liz-Marzán L. M.; et al. Present and Future of Surface-Enhanced Raman Scattering. ACS Nano 2020, 14 (1), 28–117. 10.1021/acsnano.9b04224. 31478375 PMC6990571

[ref10] Schmidt M. M.; Brolo A. G.; Lindquist N. C. Single-Molecule Surface-Enhanced Raman Spectroscopy: Challenges, Opportunities, and Future Directions. ACS Nano 2024, 10.1021/acsnano.4c09483. 39258860

[ref11] Luo S. H.; Hoff B. H.; Maier S. A.; de Mello J. C. Scalable Fabrication of Metallic Nanogaps at the Sub-10 nm Level. Adv. Sci. 2021, 8 (24), 2102756 10.1002/advs.202102756. PMC8693066 34719889

[ref12] Chen Y. K.; Li H.; Chen J. M.; Li D.; Zhang M. Y.; Yu G. H.; Jiang L.; Zong Y.; Dong B.; Zeng Z. F.; et al. Self-generating nanogaps for highly effective surface-enhanced Raman spectroscopy. Nano Res. 2022, 15 (4), 3496–3503. 10.1007/s12274-021-3924-8.

[ref13] Im H.; Bantz K. C.; Lee S. H.; Johnson T. W.; Haynes C. L.; Oh S. H. Self-Assembled Plasmonic Nanoring Cavity Arrays for SERS and LSPR Biosensing. Adv. Mater. 2013, 25 (19), 2678–2685. 10.1002/adma.201204283. 23436239

[ref14] Heo C. J.; Jeon H. C.; Lee S. Y.; Jang S. G.; Cho S.; Choi Y.; Yang S. M. Robust plasmonic sensors based on hybrid nanostructures with facile tunability. J. Mater. Chem. 2012, 22 (28), 13903–13907. 10.1039/c2jm31958f.

[ref15] Fan M. K.; Andrade G. F. S.; Brolo A. G. A review on recent advances in the applications of surface-enhanced Raman scattering in analytical chemistry. Anal. Chim. Acta 2020, 1097, 1–29. 10.1016/j.aca.2019.11.049. 31910948

[ref16] Li Q. Q.; Huo H. Q.; Wu Y.; Chen L. L.; Su L. C.; Zhang X.; Song J. B.; Yang H. H. Design and Synthesis of SERS Materials for In Vivo Molecular Imaging and Biosensing. Adv. Sci. 2023, 10 (8), 2202051 10.1002/advs.202202051. PMC10015885 36683237

[ref17] Terry L. R.; Sanders S.; Potoff R. H.; Kruel J.; Jain M.; Guo H. Y. Applications of surface-enhanced Raman spectroscopy in environmental detection. Anal Sci. Adv. 2022, 3 (3–4), 113–145. 10.1002/ansa.202200003. 38715640 PMC10989676

[ref18] Cailletaud J.; De Bleye C.; Dumont E.; Sacré P. Y.; Netchacovitch L.; Gut Y.; Boiret M.; Ginot Y. M.; Hubert P.; Ziemons E. Critical review of surface-enhanced Raman spectroscopy applications in the pharmaceutical field. J. Pharmaceut Biomed 2018, 147, 458–472. 10.1016/j.jpba.2017.06.056. 28688617

[ref19] Sun Y. J.; Lou D. Y.; Liu W.; Zheng Z. K.; Chen X. D. SERS Labels for Optical Anticounterfeiting: Structure, Fabrication, and Performance. Adv. Opt Mater. 2023, 11 (6), 2201549 10.1002/adom.202201549.

[ref20] Han X. X.; Rodriguez R. S.; Haynes C. L.; Ozaki Y.; Zhao B. Surface-enhanced Raman spectroscopy. Nat. Rev. Method Prime 2021, 1 (1), 87 10.1038/s43586-021-00083-6.

[ref21] Sloan-Dennison S.; Wallace G. Q.; Hassanain W. A.; Laing S.; Faulds K.; Graham D. Advancing SERS as a quantitative technique: challenges, considerations, and correlative approaches to aid validation. Nano Converg 2024, 11 (1), 33 10.1186/s40580-024-00443-4. 39154073 PMC11330436

[ref22] Natan M. J. Concluding remarks - Surface enhanced Raman scattering. Faraday Discuss. 2006, 132, 321–328. 10.1039/b601494c. 16833126

[ref23] Porter M. D.; Granger J. H. Surface-enhanced Raman scattering II: concluding remarks. Faraday Discuss. 2017, 205, 601–613. 10.1039/C7FD00206H. 29177326

[ref24] Dick L. A.; McFarland A. D.; Haynes C. L.; Van Duyne R. P. Metal film over nanosphere (MFON) electrodes for surface-enhanced Raman spectroscopy (SERS): Improvements in surface nanostructure stability and suppression of irreversible loss. J. Phys. Chem. B 2002, 106 (4), 853–860. 10.1021/jp013638l.

[ref25] Fredriksson H.; Alaverdyan Y.; Dmitriev A.; Langhammer C.; Sutherland D. S.; Zaech M.; Kasemo B. Hole-mask colloidal lithography. Adv. Mater. 2007, 19 (23), 4297–4302. 10.1002/adma.200700680.

[ref26] Syrenova S.; Wadell C.; Langhammer C. Shrinking-hole colloidal lithography: self-aligned nanofabrication of complex plasmonic nanoantennas. Nano Lett. 2014, 14 (5), 2655–2663. 10.1021/nl500514y. 24697350

[ref27] Kubala P.; Batys P.; Barbasz J.; Weronski P.; Ciesla M. Random sequential adsorption: An efficient tool for investigating the deposition of macromolecules and colloidal particles. Advances in colloid and interface science 2022, 306, 102692 10.1016/j.cis.2022.102692. 35753239

[ref28] Stefaniuk T.; Wrobel P.; Trautman P.; Szoplik T. Ultrasmooth metal nanolayers for plasmonic applications: surface roughness and specific resistivity. Appl. Opt. 2014, 53 (10), B237–B241. 10.1364/AO.53.00B237. 24787210

[ref29] Duan H. G.; Hu H. L.; Kumar K.; Shen Z. X.; Yang J. K. W. Direct and Reliable Patterning of Plasmonic Nanostructures with Sub-10-nm Gaps. ACS Nano 2011, 5 (9), 7593–7600. 10.1021/nn2025868. 21846105

[ref30] Wang X. J.; Zhu X. P.; Chen Y. Q.; Zheng M. J.; Xiang Q.; Tang Z. X.; Zhang G. H.; Duan H. G. Sensitive Surface-Enhanced Raman Scattering Detection Using On-Demand Postassembled Particle-on-Film Structure. ACS Appl. Mater. Interfaces 2017, 9 (36), 31102–31110. 10.1021/acsami.7b08818. 28832109

[ref31] Li M.; Xun K. X.; Zhu X. A.; Liu D.; Liu X.; Jin X. S.; Wu M. L. Research on AFM tip-related nanofabrication of two-dimensional materials. Nanotechnol Rev. 2023, 12 (1), 20230153 10.1515/ntrev-2023-0153.

[ref32] Zhang P. P.; Wu J.; Wang S.; Fang J. H. Fabrication of triangular Au/Ag nanoparticle arrays with sub-10 nm nanogap controlled by flexible substrate for surface-enhanced Raman scattering. Nanotechnology 2023, 34 (1), 015302 10.1088/1361-6528/ac9688. 36179661

[ref33] Antosiewicz T. J.; Apell S. P.; Zach M.; Zoric I.; Langhammer C. Oscillatory optical response of an amorphous two-dimensional array of gold nanoparticles. Phys. Rev. Lett. 2012, 109 (24), 247401 10.1103/PhysRevLett.109.247401. 23368376

[ref34] Harris N.; Blaber M. G.; Schatz G. C. Optical Properties of Metal Nanoparticles. In Encyclopedia of Nanotechnology, Bhushan B., Ed.; Springer: Netherlands, 2016; pp 3027–3048.

[ref35] Trivedi R.; Thomas A.; Dhawan A. Full-wave electromagentic analysis of a plasmonic nanoparticle separated from a plasmonic film by a thin spacer layer. Opt Express 2014, 22 (17), 19970–19989. 10.1364/OE.22.019970. 25321207

[ref36] Zhang C.; Li D. Y.; Zhang G. D.; Wang X. J.; Mao L.; Gan Q.; Ding T.; Xu H. X. Switching plasmonic nanogaps between classical and quantum regimes with supramolecular interactions. Sci. Adv. 2022, 8 (5), eabj9752 10.1126/sciadv.abj9752. 35119919 PMC8816333

[ref37] Czajkowski K. M.; Bancerek M.; Antosiewicz T. J. Multipole analysis of substrate-supported dielectric nanoresonator metasurfaces via the T-matrix method. Phys. Rev. B 2020, 102 (8), 085431 10.1103/PhysRevB.102.085431.

[ref38] Hu H. T.; Lu X.; Chen K.; Yan Z. D.; Cai P. G.; Tang C. J. Plasmonic Fano-type nanocavity for double resonance enhanced SERS and optical sensing. Opt. Commun. 2022, 502, 127441 10.1016/j.optcom.2021.127441.

[ref39] Tuyen L. D.; Liu A. C.; Huang C. C.; Tsai P. C.; Lin J. H.; Wu C. W.; Chau L. K.; Yang T. S.; Minh L. Q.; Kan H. C.; et al. Doubly resonant surface-enhanced Raman scattering on gold nanorod decorated inverse opal photonic crystals. Opt Express 2012, 20 (28), 29266–29275. 10.1364/OE.20.029266. 23388752

[ref40] Nugroho F. A. A.; Switlik D.; Armanious A.; O’Reilly P.; Darmadi I.; Nilsson S.; Zhdanov V. P.; Höök F.; Antosiewicz T. J.; Langhammer C. Time-Resolved Thickness and Shape-Change Quantification using a Dual-Band Nanoplasmonic Ruler with Sub-Nanometer Resolution. ACS Nano 2022, 16 (10), 15814–15826. 10.1021/acsnano.2c04948. 36083800 PMC9620406

[ref41] Mortazavi D.; Kouzani A. Z.; Kaynak A.; Duan W. Developing Lspr Design Guidelines. Prog. Electromagn Res. 2012, 126, 203–235. 10.2528/PIER12011810.

[ref42] Palik E. D. Handbook of Optical-Constants. J. Opt Soc. Am. A 1984, 1 (12), 1297–1297.

[ref43] Michota A.; Bukowska J. Surface-enhanced Raman scattering (SERS) of 4-mercaptobenzoic acid on silver and gold substrates. J. Raman Spectrosc. 2003, 34 (1), 21–25. 10.1002/jrs.928.

[ref44] Talley C. E.; Jusinski L.; Hollars C. W.; Lane S. M.; Huser T. Intracellular pH sensors based on surface-enhanced Raman scattering. Anal. Chem. 2004, 76 (23), 7064–7068. 10.1021/ac049093j. 15571360

[ref45] Capocefalo A.; Mammucari D.; Brasili F.; Fasolato C.; Bordi F.; Postorino P.; Domenici F. Exploring the Potentiality of a SERS-Active pH Nano-Biosensor. Front Chem. 2019, 7, 413 10.3389/fchem.2019.00413. 31231638 PMC6568054

[ref46] Jaworska A.; Malek K.; Kudelski A. Intracellular pH - Advantages and pitfalls of surface-enhanced Raman scattering and fluorescence microscopy - A review. Spectrochimica acta. Part A, Molecular and biomolecular spectroscopy 2021, 251, 119410 10.1016/j.saa.2020.119410. 33465573

[ref47] Lin L.; Bi X. Y.; Gu Y. Q.; Wang F.; Ye J. Surface-enhanced Raman scattering nanotags for bioimaging. J. Appl. Phys. 2021, 129 (19), 191101 10.1063/5.0047578.

[ref48] Williams A.; Flynn K. J.; Xia Z. D.; Dunstan P. R. Multivariate spectral analysis of pH SERS probes for improved sensing capabilities. J. Raman Spectrosc. 2016, 47 (7), 819–827. 10.1002/jrs.4910.

[ref49] Li R.; Lv H.; Zhang X.; Liu P.; Chen L.; Cheng J.; Zhao B. Vibrational spectroscopy and density functional theory study of 4-mercaptobenzoic acid. Spectrochimica acta. Part A, Molecular and biomolecular spectroscopy 2015, 148, 369–374. 10.1016/j.saa.2015.03.132. 25913136

[ref50] Schatz G. C.; Young M. A.; Van Duyne R. P. Electromagnetic Mechanism of SERS. In Surface-Enhanced Raman Scattering: Physics and Applications, Kneipp K., Moskovits M., Kneipp H., Eds.; Springer: Berlin Heidelberg, 2006; pp 19–45.

[ref51] Zhao J.; Dieringer J. A.; Zhang X. Y.; Schatz G. C.; Van Duyne R. P. Wavelength-Scanned Surface-Enhanced Resonance Raman Excitation Spectroscopy. J. Phys. Chem. C 2008, 112 (49), 19302–19310. 10.1021/jp807837t.

[ref52] Chu Y. Z.; Banaee M. G.; Crozier K. B. Double-Resonance Plasmon Substrates for Surface-Enhanced Raman Scattering with Enhancement at Excitation and Stokes Frequencies. ACS Nano 2010, 4 (5), 2804–2810. 10.1021/nn901826q. 20429521

[ref53] Banaee M. G.; Crozier K. B. Mixed dimer double-resonance substrates for surface-enhanced Raman spectroscopy. ACS Nano 2011, 5 (1), 307–314. 10.1021/nn102726j. 21162550

[ref54] Nam J. M.; Oh J. W.; Lee H.; Suh Y. D. Plasmonic Nanogap-Enhanced Raman Scattering with Nanoparticles. Acc. Chem. Res. 2016, 49 (12), 2746–2755. 10.1021/acs.accounts.6b00409. 27993009

[ref55] Rousseaux B.; Baranov D. G.; Antosiewicz T. J.; Shegai T.; Johansson G. Strong coupling as an interplay of quantum emitter hybridization with plasmonic dark and bright modes. Phys. Rev. Res. 2020, 2 (3), 033056 10.1103/PhysRevResearch.2.033056.

[ref56] Le Ru E. C.; Blackie E.; Meyer M.; Etchegoin P. G. Surface enhanced Raman scattering enhancement factors: a comprehensive study. J. Phys. Chem. C 2007, 111 (37), 13794–13803. 10.1021/jp0687908.

[ref57] Bell S. E. J.; Charron G.; Cortes E.; Kneipp J.; de la Chapelle M. L.; Langer J.; Prochazka M.; Tran V.; Schlucker S. Towards Reliable and Quantitative Surface-Enhanced Raman Scattering (SERS): From Key Parameters to Good Analytical Practice. Angew. Chem. 2020, 59 (14), 5454–5462. 10.1002/anie.201908154. 31588641 PMC7154527

[ref58] Le Ru E. C.; Auguié B. Enhancement Factors: A Central Concept during 50 Years of Surface-Enhanced Raman Spectroscopy. ACS Nano 2024, 18 (14), 9773–9783. 10.1021/acsnano.4c01474. 38529815

[ref59] Fleischmann M.; Hendra P. J.; McQuillan A. J. Raman-Spectra of Pyridine Adsorbed at a Silver Electrode. Chem. Phys. Lett. 1974, 26 (2), 163–166. 10.1016/0009-2614(74)85388-1.

[ref60] Ambroziak R.; Holdynski M.; Plocinski T.; Pisarek M.; Kudelski A. Cubic Silver Nanoparticles Fixed on TiO2 Nanotubes as Simple and Efficient Substrates for Surface Enhanced Raman Scattering. Materials 2019, 12 (20), 3373 10.3390/ma12203373. 31623068 PMC6830348

[ref61] Pisarek M.; Holdynski M.; Roguska A.; Kudelski A.; Janik-Czachor M. TiO2 and Al2O3 nanoporous oxide layers decorated with silver nanoparticles—active substrates for SERS measurements. J. Solid State Electr 2014, 18 (11), 3099–3109. 10.1007/s10008-013-2375-x.

[ref62] D’Andrea C.; Bochterle J.; Toma A.; Huck C.; Neubrech F.; Messina E.; Fazio B.; Maragò O. M.; Di Fabrizio E.; Lamy de La Chapelle M.; Gucciardi P. G.; Pucci A.; et al. Optical Nanoantennas for Multiband Surface-Enhanced Infrared and Raman Spectroscopy. ACS Nano 2013, 7 (4), 3522–3531. 10.1021/nn4004764. 23530556

[ref63] Lafuente M.; Berenschot E. J. W.; Tiggelaar R. M.; Mallada R.; Tas N. R.; Pina M. P. 3D Fractals as SERS Active Platforms: Preparation and Evaluation for Gas Phase Detection of G-Nerve Agents. Micromachines-Basel 2018, 9 (2), 60 10.3390/mi9020060. 30393336 PMC6187359

[ref64] Suganami Y.; Oshikiri T.; Mitomo H.; Sasaki K.; Liu Y. E.; Shi X.; Matsuo Y.; Ijiro K.; Misawa H. Spatially Uniform and Quantitative Surface-Enhanced Raman Scattering under Modal Ultrastrong Coupling Beyond Nanostructure Homogeneity Limits. ACS Nano 2024, 18 (6), 4993–5002. 10.1021/acsnano.3c10959. 38299996 PMC10867886

[ref65] Liu X.; Shao Y.; Tang Y.; Yao K. F. Highly uniform and reproducible surface enhanced Raman scattering on air-stable metallic glassy nanowire array. Sci. Rep. 2014, 4, 5835 10.1038/srep05835. 25060646 PMC5376157

[ref66] Ho C. H.; Lee S. SERS and DFT investigation of the adsorption behavior of 4-mercaptobenzoic acid on silver colloids. Colloid Surface A 2015, 474, 29–35. 10.1016/j.colsurfa.2015.03.004.

[ref67] Azziz A.; Safar W.; Xiang Y.; Edely M.; Lamy de la Chapelle M. Sensing performances of commercial SERS substrates. J. Mol. Struct. 2022, 1248, 131519 10.1016/j.molstruc.2021.131519.

[ref68] Wrobel P.; Stefaniuk T.; Trzcinski M.; Wronkowska A. A.; Wronkowski A.; Szoplik T. Ge Wetting Layer Increases Ohmic Plasmon Losses in Ag Film Due to Segregation. ACS Appl. Mater. Interfaces 2015, 7 (17), 8999–9005. 10.1021/acsami.5b01471. 25871505

[ref69] Witkowski M.; Starowicz Z.; Zieba A.; Adamczyk-Cieslak B.; Socha R. P.; Szawcow O.; Kolodziej G.; Haras M.; Ostapko J. The atomic layer deposition (ALD) synthesis of copper-tin sulfide thin films using low-cost precursors. Nanotechnology 2022, 33 (50), 505603 10.1088/1361-6528/ac9065. 36075187

[ref70] Witkowski M.; Krolikowska A.; Cukras J.; Dzwolak W. Hidden Dynamics of Noble-metal-bound Thiol Monolayers Revealed by SERS-monitored Entropy-driven Exchange of Cysteine Isotopologues. Appl. Surf. Sci. 2023, 623, 156985 10.1016/j.apsusc.2023.156985.

