# Supplementary material for: Nanogap-Engineered Core–Shell-Like Nanostructures for Comprehensive SERS Analysis
Source: ACS Appl Mater Interfaces. 2025 Apr 3;17(15):23076–93. doi: 10.1021/acsami.5c00716 (PMC12012730; doi:10.1021/acsami.5c00716)
Supplement: Supplementary file 1 — am5c00716_si_001.pdf [file am5c00716_si_001.pdf]

Supporting Information for: Nanogap-Engineered Core–Shell-Like Nanostructures for Comprehensive SERS Analysis

Mihai C. Suster†¶, Aleksandra Szymańska‡†¶, Tomasz J. Antosiewicz†, Agata Królikowska‡*, and Piotr Wróbel†*

†Faculty of Physics, University of Warsaw, Pasteura 5, 02-093 Warsaw, Poland
‡Faculty of Chemistry, University of Warsaw, Pasteura 1, 02-093 Warsaw, Poland

¶Contributed equally to this work

E-mail: akrol@chem.uw.edu.pl; Piotr.Wrobel@fuw.edu.pl

S1. FABRICATION PROCEDURE OF CSLNs

Amorphous arrays of Core–Shell-Like Nanostructures (CSLNs) were prepared by the self-assembly of Dielectric NanoSpheres (DNSs) of four distinct diameters (*d*): 60 nm, 100 nm, 200 nm, and 300 nm. Ge films of 1.5 nm thickness and Ag films of various thicknesses (5 nm - 50 nm in height (*h*)) were deposited over the DNSs by the ePVD (electron beam physical vapor deposition) technique. A 5 nm thick layer of Au was also evaporated on selected substrates. The following fabrication process was confirmed to operate on many typical solid supports used in nanomanufacturing, including microscopic soda-lime glass slides and wafers made of silicon, sapphire, GaAs, *etc.* Here, the term 'support' refers to a bare solid substrate, while 'substrate' is used to describe a solid support covered with plasmonic nanostructures.

One of the most challenging aspects of working with colloidal nanoparticles is ensuring their homogeneous distribution across the entire surface of a substrate with no signs of oligomerization. Here, three distinct procedures were applied to address this issue. First, the DNSs coated with negatively charged surface functionalities were selected, which prevents agglomeration of DNSs in suspension. Second, DNSs suspensions of very low concentrations were used to minimize the direct contact between two or more support-attached DNSs. Third, before deposition of the DNSs, the glass supports were coated with a few nanometers thick layer of poly(diallyldimethylammonium chloride) (PDDA). This cationic polymer ensures no migration of the DNSs across the substrate surface due to the electrostatic attraction between the polymer and the DNSs. In addition to these three factors, the development of a reliable cleaning procedure (see below for details) contributed to the spatial uniformity of the CSLNs. The effectiveness of this approach is demonstrated in the scanning electron microscopy (SEM) images in Figure S1, which display a large-area spatial homogeneity for the metal coated DNSs of two different diameters. The mean distance between the DNSs deposited on the support is concentration-controlled. In addition, the inset in Figure S1a

illustrates the macroscopic uniformity of the entire substrate, following the fabrication protocol optimized for surface-enhanced Raman scattering (SERS) analysis.

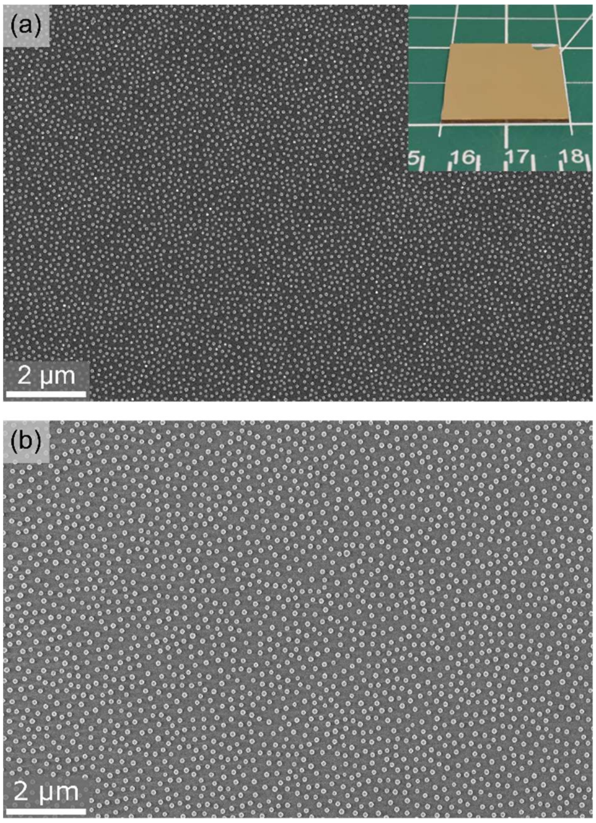

Figure S1: SEM micrographs confirming high spatial uniformity of CSLN substrates over a large area. The proposed fabrication procedure makes it possible to achieve presented homogeneity across an area of >6 cm^2. (a) 60 nm DNSs, (b) 100 nm DNSs, both covered with 1.5 nm Ge and 20 nm Ag. Inset: photograph of a 2.5 cm × 2.5 cm substrate showing the uniformity of the CSLNs composed of 60 nm DNS, coated with 1.5 nm Ge, 40 nm Ag, and 5 nm Au (final geometry optimized for SERS performance). The absence of the metal layer in the upper right corner of the substrate is due to the metal clips holding and masking this area of a sample during the evaporation process. The scale in the inset is marked in centimeters.

Results presented in this paper were acquired using microscopic glass slides as supports, cut into 2.5 cm × 2.5 cm pieces to enable optical transmission measurements, as well as to reduce the overall manufacturing cost. The cleaning process of the support consisted of two stages: macroscopic and microscopic. The purpose of the first one is to remove any dust, fingerprints, or other relatively large objects from the surface by thoroughly rinsing with ethanol and deionized water. The second stage eliminates further contaminants at the molecular scale by oxygen plasma treatment with the plasma cleaner. This cleaning procedure should be repeated two or more times, depending on the type and condition of the solid support. When performed properly, the resulting cleanliness and wettability

allow various liquids to form a uniform film across the entire surface of the glass. In the next step, an adequate amount of 0.2 wt% PDDA solution suspended in water (typically 1 ml) was evenly pipetted over the entire surface of the support, ensuring no overspill. Excess PDDA was then rinsed off with pure water and blown away by a stream of compressed nitrogen or argon gas. After applying such an interlayer, the DNSs suspended in water were pipetted onto the samples in the same manner as the PDDA.

Finally, various types of metal coatings were evaporated onto the DNSs covered supports using a PVD75 Kurt J. Lesker ePVD system, with evaporation rates ranging from 0.3 to 1 Å/s. The total thickness of the evaporated layers and multilayers shown in this paper varied from 6.5 nm to 56.5 nm. Exemplary geometric parameters, such as the height (*h*) and width of the metal cap, are shown in SEM images in Figure S2, presenting a CSLN composed of a 300 nm DNS diameter (*d*) and a 100 nm thick Ag layer. An aperture surrounding the bottom of each nanosphere is also clearly visible. To achieve both uniform and smooth coverage over the DNSs, it is strongly recommended to form a wetting layer prior to evaporation of the plasmonic metal (here: 1.5 nm of Ge in the case of Ag coating). This is important, particularly for thin Ag layers (< 20 nm), as they usually do not form a continuous film when deposited alone.

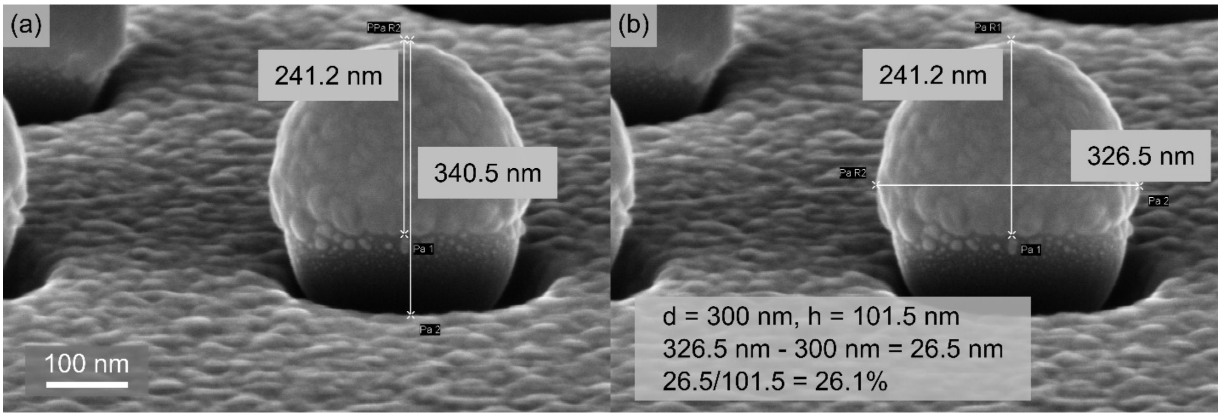

Figure S2: SEM micrographs presenting the details of CSLN geometry, such as the height and width of the metal cap, as well as the characteristic aperture for a nanostructure consisting of a 300 nm DNS diameter, 1.5 nm Ge, and 100 nm Ag layer. The nominal values of a diameter (*d*), and an evaporated metal height (*h*) are given in the legend, together with a calculated shell thickness on the side of the sphere, and a determined shell-to-height ratio.

The effect of a wetting Ge layer versus its absence on the optical response and geometric features of the nanostructures is presented in Figure S3.

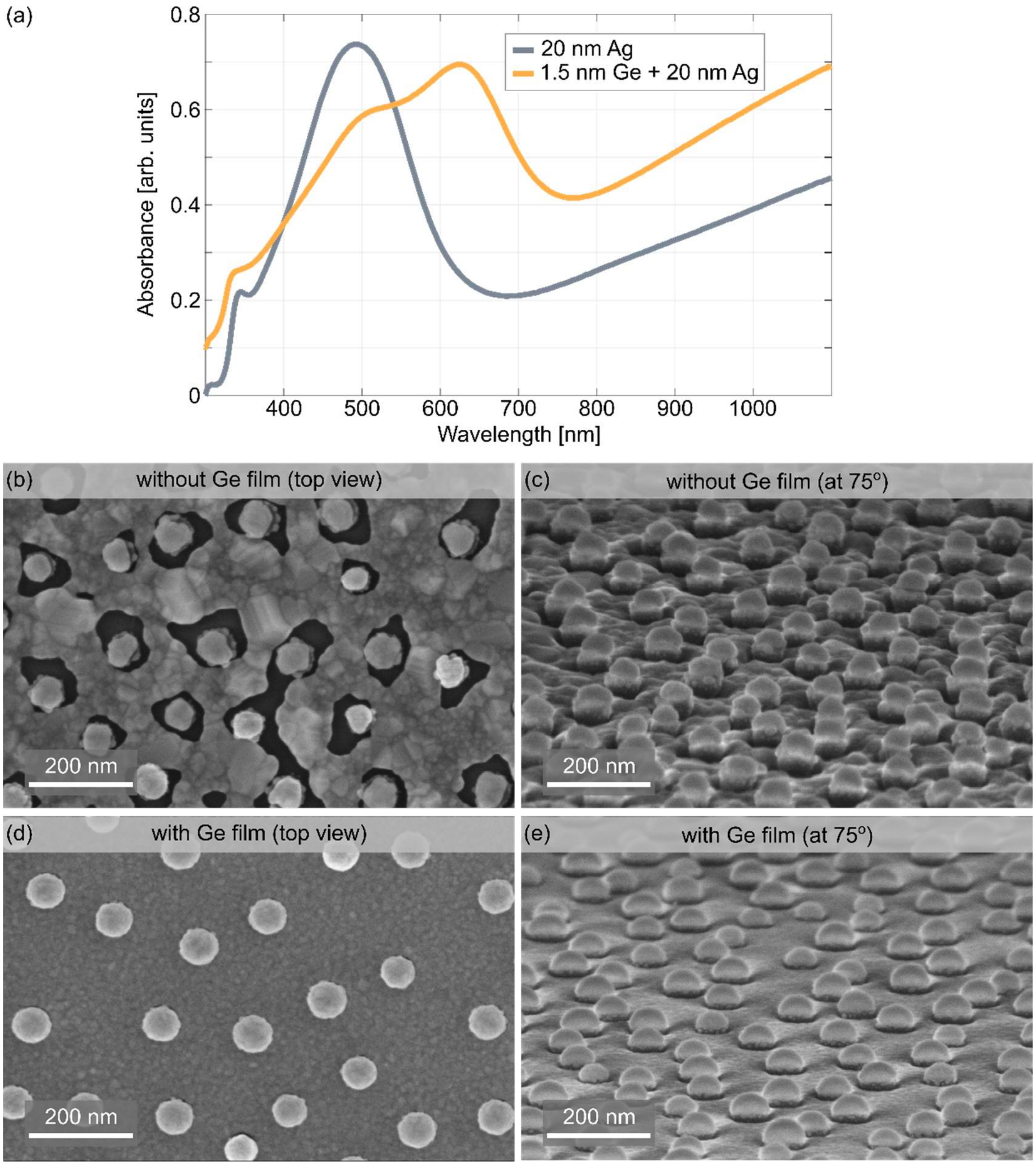

Figure S3: (a) Absorbance spectra of Ag coated CSLNs with and without a 1.5 nm Ge wetting layer (*d* = 60 nm, *h* = 20 nm), (b)-(d) SEM micrographs illustrating the effect of a wetting layer on topography and structural details of the CSLNs. Images (b) and (d) were taken at 90° to the substrate plane, while images (c) and (d) were taken at 75° angle.

When an Ag film is deposited directly onto the glass support covered with DNSs (SEM images in Figure S3b and c) it creates a rough semi-continuous coating, with visible large grains. Due to high curvature of the DNS, which further reduces its wettability, Ag does not form a well-adhering cap over the DNS. Instead, it creates a single silver nanoparticle of a poorly (and differently) defined shape on top of each DNS.

This is further confirmed by the absorbance spectrum, which for this type of structure resembles the optical response of a silver nanoparticle (Figure S3a; gray curve). However, when a wetting layer is deposited underneath the Ag film (SEM images in Figure S3d and e), the grains become barely visible, the continuity of the layer is greatly improved, and the DNSs metallic coating adheres properly. This improvement is also reflected in the change of absorbance spectrum (Figure S3a; orange curve), which shows three maxima typical of core–shell-like nanostructures, with the highest intensity of the plasmon mode associated with electromagnetic (EM) field enhancement in the nanogap region. This demonstrates that ensuring good wettability of the substrate is essential for this architecture to obtain CSLNs geometry that will generate nanogap mode enhancement.

Various techniques have been developed for fabrication of sub-10 nm nanogaps suitable for applications in SERS analysis. A comparison of these state-of-the-art methods with the approach proposed here, including the advantages and disadvantages of each technique, is summarized in Table S1.

Table S1: Comparison of sub-10 nm nanogap fabrication methods for SERS applications

**Technique**	**Minimum gap size**	**Pros**	**Cons**	**Ref.**
Electron beam lithography	~5 nm	High precision & aspect ratio<br>Complex patterning<br>Material versatility	Low throughput<br>Slow processing<br>Small area<br>Multistep process<br>High cost of equipment &<br>Operation limited to electron-sensitive resists	[1, 2]
NanoSphere -on-mirror (NSOM)	<1 nm	Simple and cost-effective fabrication<br>Large-area scalable & tunable<br>Material versatility	Gap size limited by the material thickness<br>For gap-forming dielectrics – gaps not accessible for molecules<br>Limited structural complexity<br>Nanoparticle positioning challenges<br>Precision dependent on nanosphere quality	[3]

Tip-based nanofabrication	<5 nm	Nanogaps/grooves with arbitrary pattern<br>Material versatility<br>Mask- and resist-free<br>Single-step fabrication<br>High precision and resolution	Slow processing speed<br>Tip wear and degradation<br>Limited depth control and uniformity<br>Surface interaction challenges affects reproducibility<br>Limited scalability	[4-6]
Nanoskiving	<10 nm	Simple & cost-effective<br>Mask-free Scalable High aspect ratio Material versatility	Precision limited control over individual nanostructure placement<br>Requires highly controlled slicing process<br>Mechanical artifacts and edge roughness<br>Requires embedding in epoxy, which may affect some applications<br>Prone to mechanical deformation	[7, 8]
Nanosphere lithography (NSL)	~5 nm	Cost-effective & scalable fabrication<br>High throughput<br>Material versatility<br>Tunable nanostructure size and periodicity	Limited geometry<br>Defects, self-aggregation & disordering issues in the nanosphere mask<br>Nanogap size limited by sphere diameter<br>Surface treatment required for adhesion	[9]
Anodic aluminum oxide	~10 nm	Large-area patterning<br>Low-cost and scalable<br>High order and aspect ratio<br>Tunability of multiple geometrical parameters<br>Compatibility with various substrates<br>Tunability of multiple geometrical parameters	Limited geometry and material<br>Challenging pore size reduction below 10 nm Limited lateral structure control and uniformity issues<br>Template removal challenges	[10]

Proposed work	<10 nm	Large-area<br>Cost-effective & Simple fabrication<br>Limited self-aggregation (nanospheres <300 nm)<br>Material versatility<br>Tunability of multiple geometrical parameters<br>Temporal stability	Limited geometry options<br>Surface treatment required for adhesion<br>Precision dependent on nanosphere quality (*e.g.*, size distribution)<br>Self-aggregation issues (nanospheres >300 nm)

S2. CORE–SHELL-LIKE OPTICAL RESPONSE

Figure S4 shows the FDTD calculated scattering, absorption, and extinction cross-sections of fully metal-coated latex-Ag core–shell (CS) nanospheres placed on a semi-infinite glass support. This system, consisting of a latex core sphere with a diameter of 60 nm and an Ag shell thickness of 20 nm, exhibits three distinct spectral features associated with plasmonic resonances present in the extinction cross-section curve.

The most prominent maximum at about 450 nm corresponds to the symmetric dipolar mode with its corresponding electric field distribution shown in Figure S4d. The maximum at 350 nm is associated with the antisymmetric mode, whose EM field distribution is concentrated at the inner core–shell interface (Figure S4b). The intermediate resonance occurring at 380 nm is attributed to a higher-order quadrupole mode (see Figure S4c for the electric field distribution) and can be observed in case of the large overall size (including both dielectric core and metal shell) of the nanoparticle (*i.e.,* 100 nm), while it is absent in the spectra of smaller CS nanoparticles.

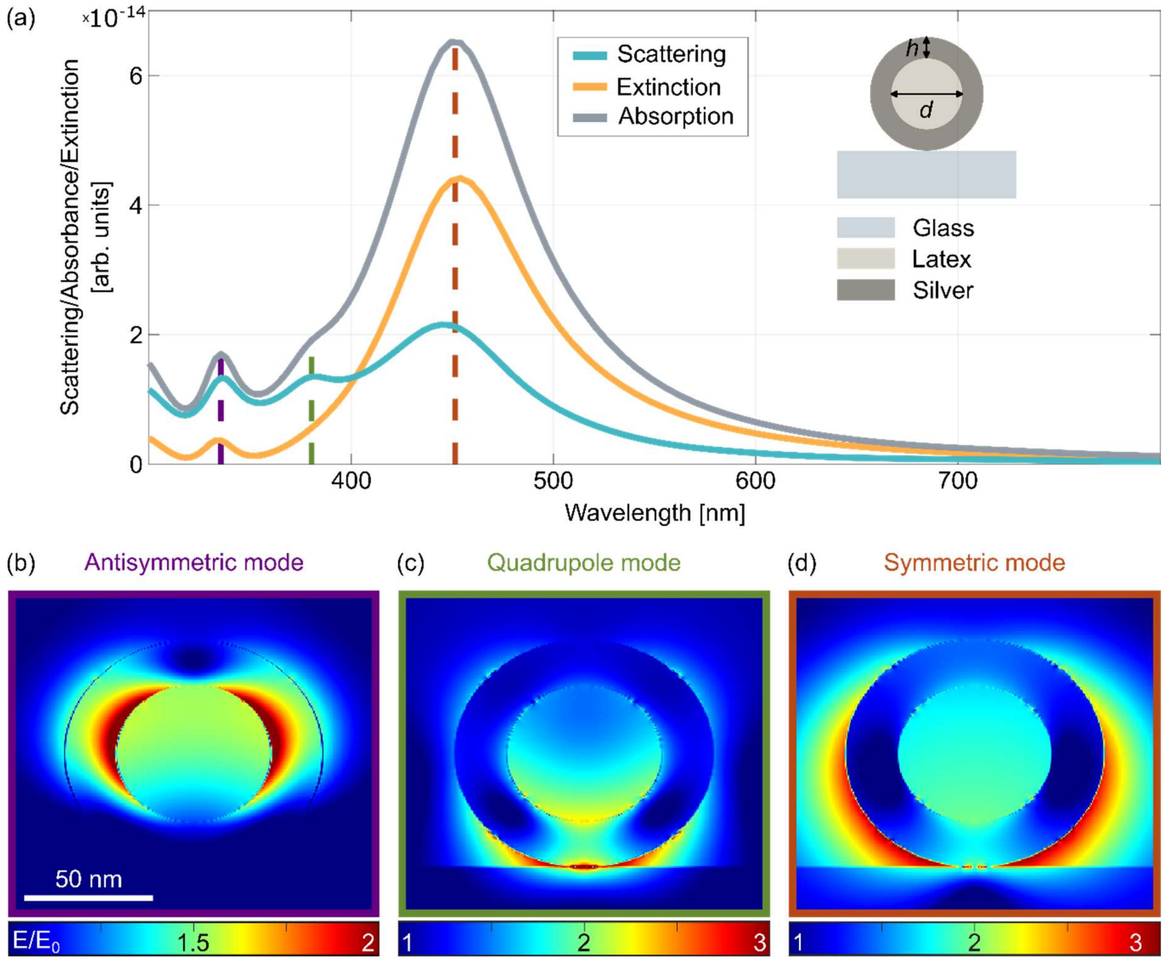

Figure S4: (a) FDTD-calculated extinction, scattering, and absorption cross-sections of a fully metal-coated core–shell nanosphere with a 60 nm latex core (modeled with a refractive index of SiO_2), and an Ag shell of thickness *h* = 20 nm on a glass substrate (see the model geometry on the right). (b)-(d) Electric field distributions for the structure depicted in (a), corresponding to the plasmonic modes calculated at the resonant wavelengths indicated by the color-coded dashed lines in panel (a).

The occurrence of the symmetric and antisymmetric modes is characteristic of core–shell nanospheres. Their presence in the extinction spectrum can be distinguished by analyzing their spectral positions as a function of shell thickness (see Figure S5 for the results of FDTD simulations). In the case of a 5 nm thick metal shell, these two resonances are separated by a few hundred nanometers (see a red curve in Figure S5). Increasing the thickness of the metal shell leads to a blue shift of the symmetric mode and a red shift of the antisymmetric mode towards the spectral position corresponding to the dipolar response of a solid metal nanoparticle.

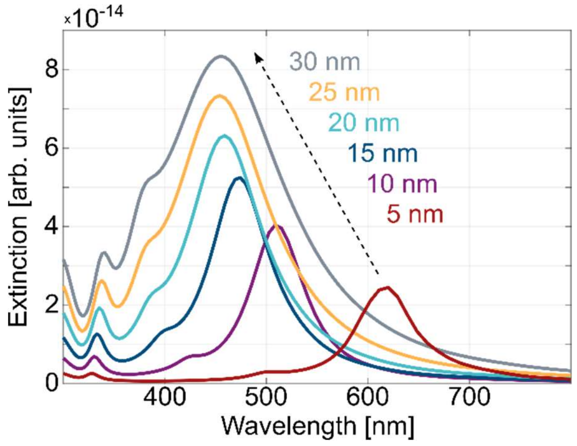

Figure S5: FDTD-calculated extinction cross-sections of the fully metal-coated core–shell nanosphere, composed of a 60 nm diameter (*d*) latex core, and Ag shell thickness (*h*) varying from 5 nm to 30 nm. An arrow indicates a blue shift of the symmetric plasmonic mode with increasing shell thickness.

The two analyzed resonances do not overlap but remain separated for the thickest simulated metal layer (30 nm in Figure S5; gray curve), while the quadrupole mode resonance appearing in between them becomes more pronounced with the growing thickness of the metal shell. The characteristic multiresonance optical response (Figure 2a in the main manuscript), along with the electric field distributions at the corresponding resonances (Figure 2b-d) and the dependence of resonance positions on shell thickness in the proposed CSLN geometry (Figure 3a and b), confirm their similarity to fully coated CS nanoparticles. Additionally, the presence of the nanogap enables control of light in the strongly subwavelength region, surpassing the capabilities of classical CS systems.

The FDTD model employed in this study, although developed using experimental data – including ellipsometrically measured optical parameters of the materials, SEM-measured geometrical parameters, and optimized to match spectroscopically measured spectral response – presents challenges due to the complexity of the geometry at the sub-nanometer scale. Modeling the macroscopic optical response of such fine nanostructures requires simplifications to at least qualitatively approximate and understand the system's behavior.

The idealization of the fabricated structure does not account for several key experimental factors:

1. Size distribution of the dielectric nanospheres at a given metal thickness, which influences the nanogap size distribution.

2. Variations in the real interparticle distance, which in simulations are modeled with a single value treated as the average distance. Close-packed nanoparticles may induce resonance shifts and modifications of the enhancement factor (EF) due to the strong near-field interactions

3. Surface roughness of both the flat metal layer and the metallic cap, which may cause non- resonant light scattering, reducing the spectral contrast and broadening plasmonic resonances.

4. The exact cap geometry, which in experiments often exhibits some porosity, forming smaller nanoparticles at the rim of the dielectric spheres. In contrast, the model assumes a continuous semi-elliptical cap with a rectangular edge.

5. Differences in illumination conditions – the experiment employs incoherent, unpolarized light from a thermal source, while the simulations use a coherent, linearly polarized plane wave.

However, despite these simplifications/approximations, the adopted model successfully reproduces multiresonance spectral features, accurately predicting the position and shape of the two resonances at the shorter wavelengths. Unfortunately, only qualitative agreement is observed for the position and intensity of the nanogap plasmonic resonance, which is inherently highly sensitive to nanoscale features and proper phase relations between incident and scattered light. Here, the latter is only coarsely approximated using periodic boundary conditions and thus the agreement is worse than for the other two resonances.

To identify the most critical factors contributing to these discrepancies, we analyzed the impact of key parameters on the nanogap resonance's position and peak intensity (Figure S6), including:

- Ag layer nominal thickness on top of the DNS and a substrate (h)
- Diameter of the dielectric nanosphere (d)
- Size of the simulation unit cell which determines average center-to-center DNS distance to approximate interparticle coupling. This approach is, however, only able to coarsely represent the amorphous distribution of particles. Its biggest limitation is the need to avoid lattice resonances which are never present in an amorphous array, hence subwavelength periods are used and thus near-field coupling is overestimated above the value present in the experimental amorphous array.
- Thickness of the Ag layers at the side of the DNS (q).

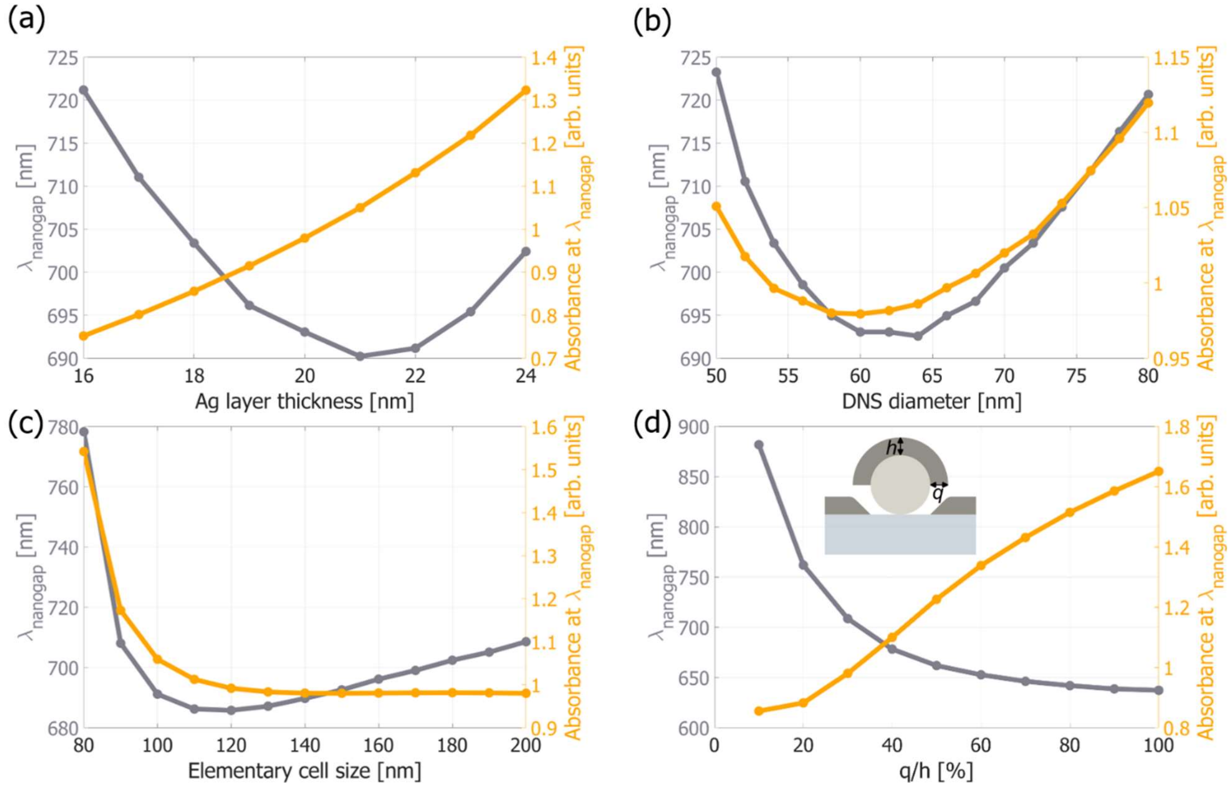

Figure S6: Influence of the FDTD model parameters on the nanogap resonance position (λ_{nanogap}) and its peak amplitude for varying: (a) Ag layer thickness (*h*), (b) DNS diameter (*d*), (c) elementary cell size describing center-to-center interparticle distance, and (d) thickness of the metal cap a the side of dielectric sphere shown as percentage of the Ag layer thickness on top (*q*/*h*; see the model in the inset). Calculations showing the influence of each of these variables were conducted for fixed values of: *h* = 20 nm, *d* = 60 nm, and elementary cell size of 150 nm, all of which are used as nominal values in the optimized model.

Our FDTD analysis reveals the following trends (see Figure S6a-c):

- Ag layer thickness (*h*) and DNS diameter (*d*) influence nanogap size, leading to a blueshift of the resonance as metal thickness and DNS diameter increase (cf. Figure S6a and b). However, beyond a gap size of 7.5 nm, strong interactions between localized modes at the cap and hole edges cause a redshift of the resonance.

- The peak intensity follows a similar trend as for the resonant wavelength for changes in DNS diameter (Figure S6b), while for the increasing metal thickness, the resonance intensity rises monotonically (Figure S6a) due to greater absorption from the increased amount of lossy material.

- Interparticle distance affects resonance position (Figure S6c) – for center-to-center distances below 110 nm, strong interparticle interactions induce a rapid redshift of peak position and an increase in resonance intensity. Beyond this distance, the intensity stabilizes, and the resonance shift becomes very weak.

While these FDTD-employed parameters significantly influence the resonance position, none of them shifts the nanogap resonance peak toward its experimentally measured resonant wavelength (~620 nm for 60 nm DNS diameter and 20 nm Ag nominal thickness), indicating that they are not the primary cause of the observed discrepancy.

The most critical factor affecting the resonance position appears to be the metal cap thickness at the side of the DNS (parameter q in the model presented as inset of Figure S6d). In FDTD simulations (Figure S6d), this thickness is expressed as a percentage of the Ag layer thickness on top of the cap (h), assuming the latter matches the planar layer thickness of the substrate. In experiments, this thickness corresponds to ~30% of the nominal deposited Ag layer thickness, resulting from evaporation conditions and the geometry of the metal coated DNS-decorated substrate, and cannot be independently adjusted. The model predicts that increasing the side thickness (q) results in a rapid blueshift of the nanogap peak down to ~630 nm when the q (side thickness) matches the h (nominal Ag layer thickness on top). However, this shift is accompanied by an overestimation of the resonance amplitude – twice as high as in the experiment and thus only one spectral parameter is improved in this manner. Since such a thick side layer is not observed experimentally, this discrepancy suggests that the assumed metal distribution in the model is not the primary cause of the deviation, which takes us back to the initial discussion of adopted model simplifications.

S3. EFFECT OF A THIN GOLD COATING ON PLASMONIC CHARACTERISTICS

Motivated by the goal to improve the chemical stability and biocompatibility of substrates, we investigated the effect of a thin (5 nm) top layer of Au on the plasmonic properties of CSLNs. Figure S7 highlights the optical differences between the selected substrates with identical fabrication parameters, except for the overall thickness and/or the composition of the metal (multi)layer. The absorbance curves were collected for CSLNs consisting of 60 nm DNS coated with 1.5 nm Ge, and 20 nm (gray), 25 nm Ag (cyan) and 20 nm Ag capped by 5 nm Au (orange). Understandably, the general pattern of the absorbance curve changes slightly upon replacement of 5 nm Ag with Au (cf. cyan and orange curves). The resemblance of absorbance curves corresponding to CSLNs with a 25 nm thick Ag layer and an Ag/Au bilayer (25 nm in total) indicates that the overall metal thickness is the primary factor governing the shape and position of the plasmonic resonances in the proposed architecture. The introduction of a 5 nm thick gold layer, exhibiting higher extinction coefficients than silver, does not degrade the plasmonic resonances associated with symmetric and antisymmetric modes, preserving the optical quality of the nanostructure while significantly enhancing its chemical stability and durability.

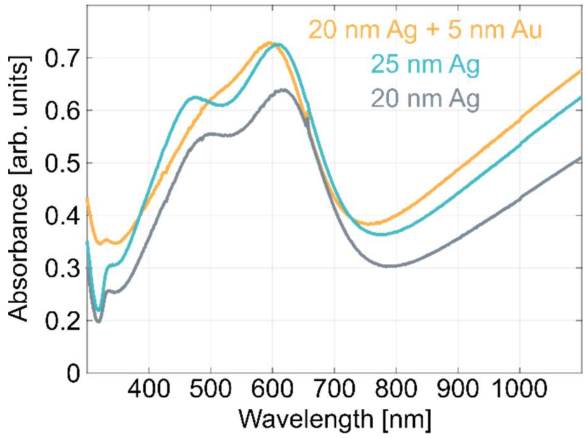

Figure S7: Effect of a 5 nm thick gold layer on the absorbance spectra of CSLNs formed by 60 nm DNSs (0.2 wt% suspension) coated with 1.5 nm Ge, and a 20 nm Ag layer compared to CSLN without Au layer and solely Ag layer of thickness 20 nm and 25 nm.

S4. PARAMETERS OF CSLN SUBSTRATES CRITICAL FOR SERS PERFORMANCE

A typical experimental SERS spectrum of *p*-mercaptobenzoic acid (pMBA) excited with a green laser (532.0 nm) for the molecular layer adsorbed on CSLN substrate fabricated using parameters optimized through the procedure described in the main manuscript, is presented in Figure S8. The

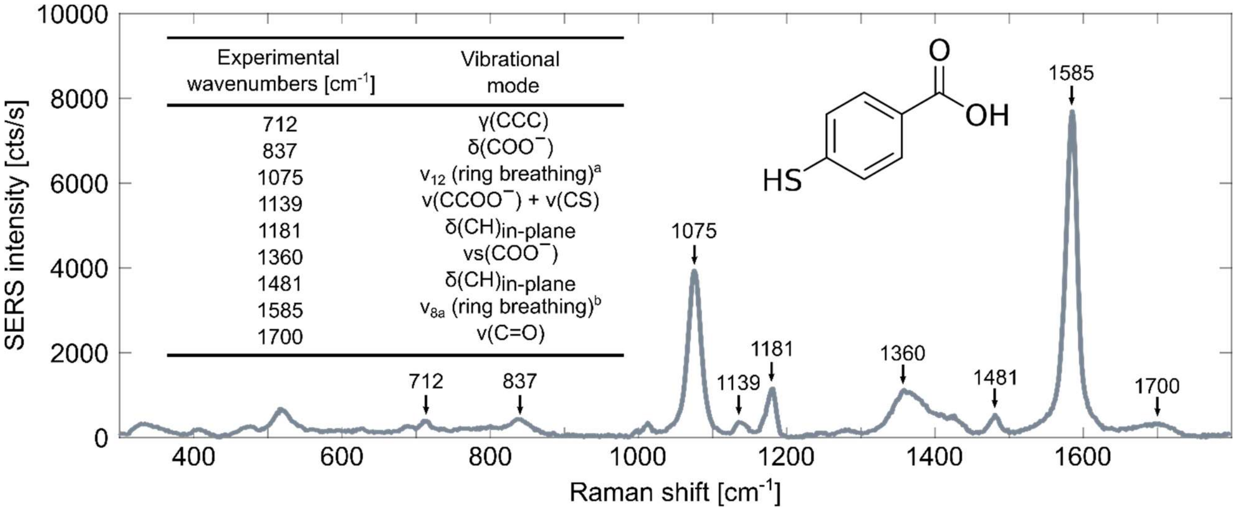

Figure S8: Typical SERS spectrum (excited with 532.0 nm laser) of pMBA SAMs grown overnight from 10^{-4} M ethanolic solution on CSLNs fabricated with 0.2 wt% of 60 nm DNSs, coated with 1.5 nm Ge, 40 nm Ag, and 5 nm Au. Inset: molecular formula of pMBA and a table comprising the most intense SERS bands of pMBA. The peak positions and vibrational assignment of major SERS bands observed for the pMBA layer are based on literature reports.[11-14] Greek symbols denote types of vibrations: γ – out-of-plane deformation, δ – bending, ν – stretching. [a and b] Wilson's notation for normal modes of benzene.

molecular formula of pMBA and complete vibrational assignment of its main SERS bands is shown in the inset of Figure S8. For a detailed discussion, the reader is referred to the "SERS performance of CSLNs substrates" section of the manuscript.

The degree of coverage of the solid support with DNSs, guided by a specific fabrication protocol, affects the efficiency of the SERS enhancement due to the simple correlation between the density of nanospheres distribution and the number of hot spots illuminated by the laser, contributing to the overall SERS signal. The results of such analysis based on SEM images for the CSLNs prepared with nanospheres of different diameters are presented in Figure S9. The degree of surface coverage by nanospheres decreases with increasing DNS diameter, reaching 17.7%, 14.9%, 13.0% and 11.6% for *d* equal to 60 nm, 100 nm, 200 nm and 300 nm, respectively. The trend observed in Figure S9 is qualitatively in good agreement with the changes in SERS intensity in Figure 6d in the main manuscript. The discrepancies are due to the fact that for DNSs of different sizes, SERS activity also depends strongly on the plasmonic properties of the substrate, with a particularly significant contribution of the nanogap mode.

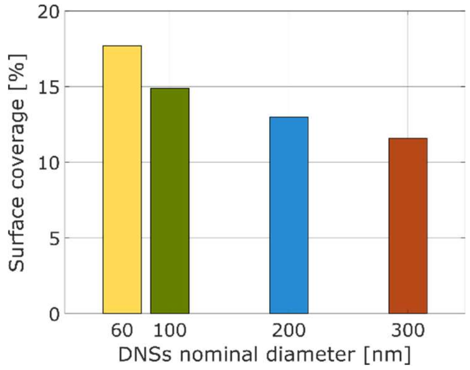

Figure S9: Changes of surface coverage determined from the analysis of SEM images (calculated as a ratio of DNSs surface area and total area of the substrate) for CSLNs fabricated with 0.2 wt% DNSs of varying nominal diameters: 60 nm, 100 nm, 200 nm, and 300 nm; each coated with 1.5 nm Ge, 40 nm Ag, and 5 nm Au. At least 55 objects were analyzed for each case.

A large part of the analysis in the main manuscript focuses on the effect of plasmonic enhancement through nanogap mode on the SERS signal. To estimate the nanogap size for different DNSs diameters, the structural analysis presented in Figure S10 was performed. SEM images taken at 75° were used to measure the distance from the edge of the aperture to the bottom border of the metal cap coating DNS, considered the most reliable evaluation of the nanogap size. The largest measurement uncertainty is observed for the geometry shown in Figure S10a, which corresponds to the smallest DNSs size and, consequently, the smallest nanogap size. In this case one can also see a slight non-uniformity around the perimeter of a given DNS.

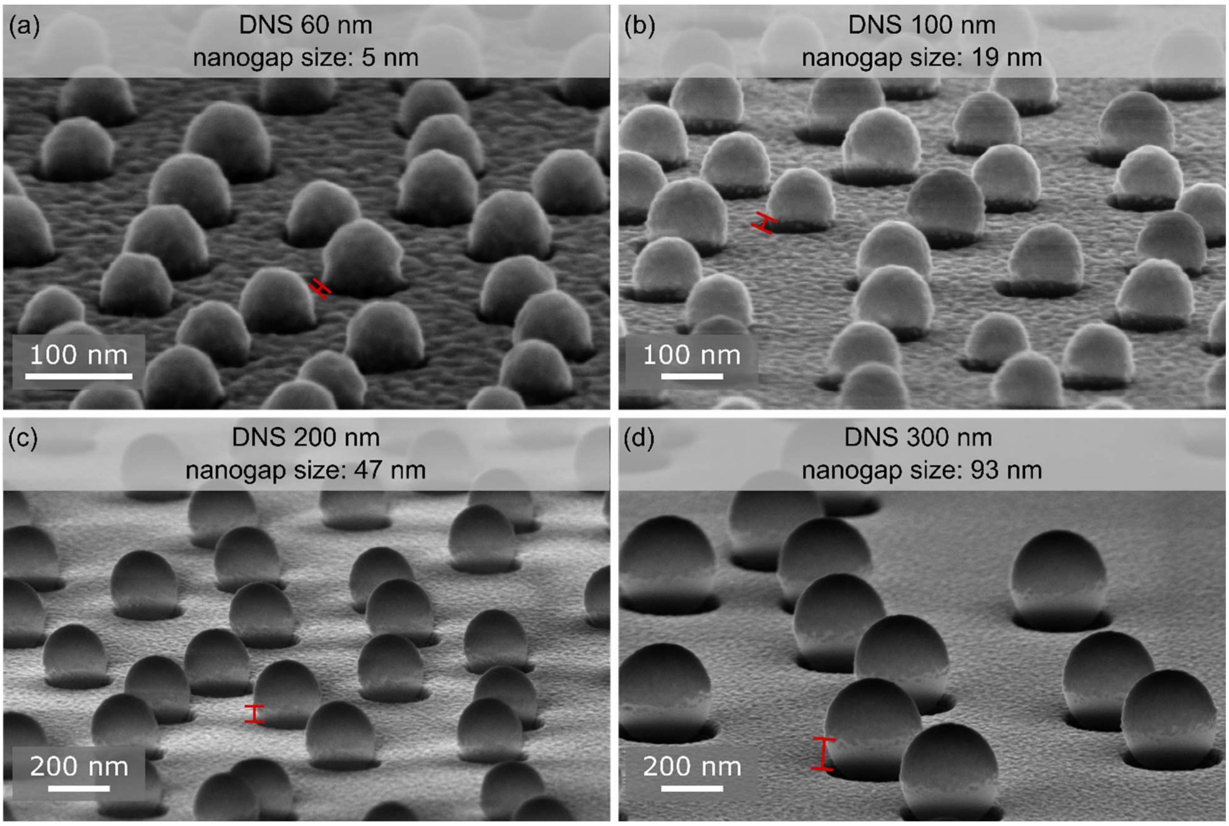

Figure S10: SEM images comparing nanogap sizes for CSLNs fabricated with 0.2 wt% DNSs of varying diameters: (a) 60 nm, (b) 100 nm, (c) 200 nm, and (d) 300 nm, each coated with 1.5 nm Ge, 40 nm Ag, and 5 nm Au. One object was analyzed for each case. Red markers indicate typical regions selected for estimating nanogap dimensions. The DNS diameters and corresponding nanogap sizes are given in the legend.

Statistical analysis of SEM images was performed to assess the size dispersion of metal-coated DNSs diameter, which impacts the relative standard deviation (RSD) of the SERS spectra. The diameters of at least 57 DNSs were determined from each SEM image, and the standard deviation (SD) for each substrate was calculated. The results presented as histograms in Figure S11 align closely with the observed RSD values of the SERS spectra for DNS of varying size and constant total metal layer thickness. Comparison of the calculated SD values (σ) for determined mean diameters (μ) (shown in Figure S11) with the data in Figure 6g in the main manuscript reveals a clear relationship between DNS size dispersion and RSD of the SERS signal. The higher the SD of metal- coated DNS diameter, the greater the RSD in SERS signal, resulting from a less uniform geometry of the DNSs comprising an amorphous array of CSLNs and thus its less well-defined plasmonic response.

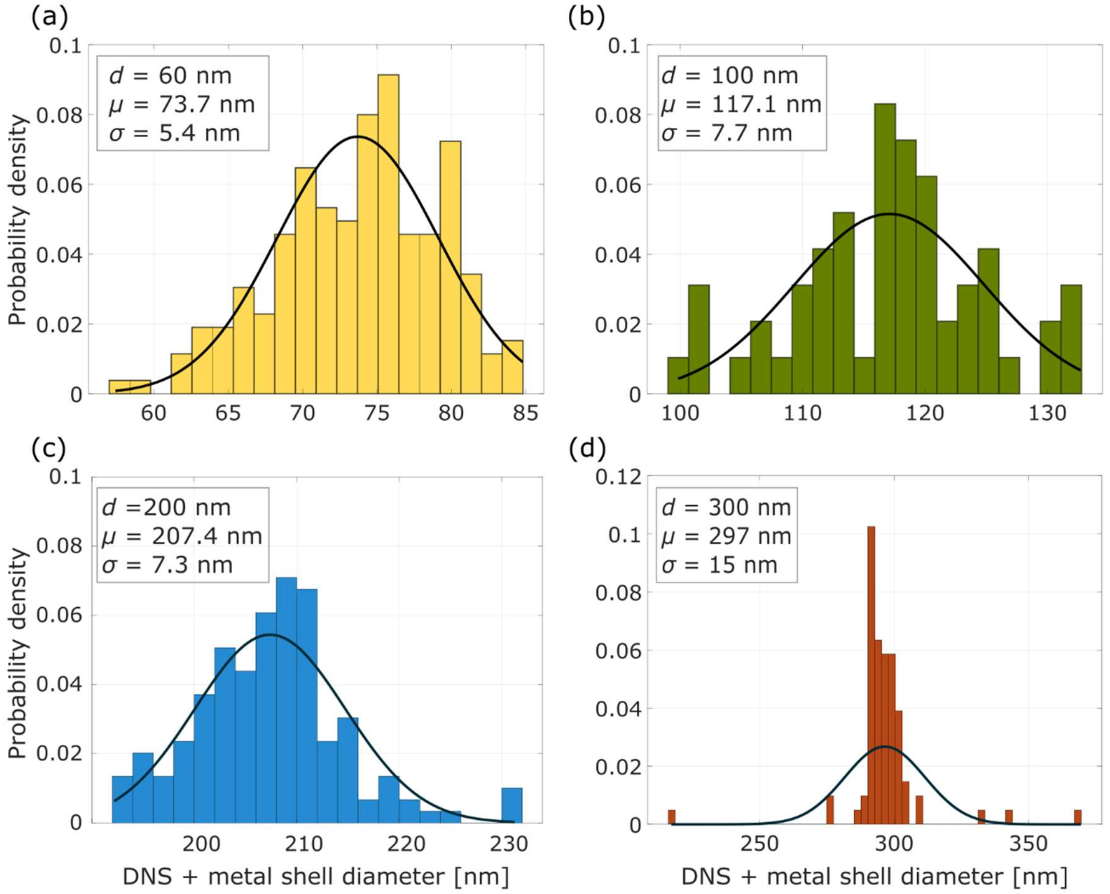

Figure S11: Size distribution histograms of DNS – for the size expressed as DNS diameter (*d*) plus Ag and Au shell total thickness, determined from the analysis of SEM images – for the CSLNs fabricated with 0.2 wt% suspensions of nanospheres with varying nominal diameter *d* (see the legend in panels (a)-(d)). The resulting SD values (σ) for such calculated mean diameters (μ) of metal-coated dielectric nanospheres are given in the legend. At least 57 objects were analyzed for each diameter of DNS, evaporated with nominally 40 nm Ag, and 5 nm Au thick metal multilayer.

The effect of DNSs suspension concentration on substrate surface coverage was also investigated, and the results obtained from the analysis of SEM images are shown in Figure S12 (the adopted procedure was identical to that for Figure S9). In this case, one can see how significant this particular parameter is for the observed SERS signal intensity (cf. Figure 6g in the main manuscript), as evidenced by an excellent agreement of the compared relationships.

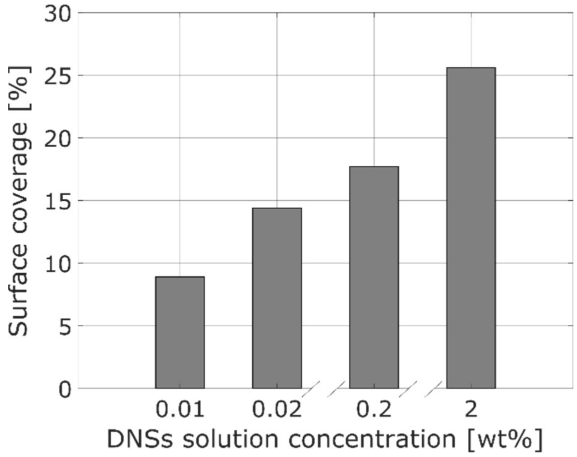

Figure S12: Changes of surface coverage determined from the analysis of SEM images (calculated as a ratio of DNSs surface area and total area of the SEM image) for CSLNs fabricated with 60 nm DNSs suspensions of varying concentration: 2, 0.2, 0.02, and 0.01 wt%; each coated with 1.5 nm Ge, 40 nm Ag, and 5 nm Au. At least 105 objects were analyzed for each case.

To study uniformity of the signal enhancement, we collected SERS maps for substrates of individual geometries with 10^{-4} M pMBA adsorbed. Figure S13 shows the results of SERS mapping with a 532 nm laser for three areas at least 1 cm apart (each scanned over an area of about 55 × 60 µm, with a 15 × 15 point grid) of the optimized CSLN substrate; i.e., fabricated with the 0.2 wt% of 60 nm DNSs, coated with 1.5 nm Ge, 40 nm Ag and 5 nm Au. The plots show the contrast illustrating the changes in intensity at the maximum for the pMBA band at 1585 cm^{-1} and its relative intensity to the mean spectrum (for 225 points) from the entire map. The selection of such parameters to evaluate their spatial distribution provided good reference to the SERS results presented in Figure 6a and b in the main manuscript. The SERS maps for the optimized CSLN substrate confirm its exceptional spatial homogeneity, both at the micro- (as visible in each individual map in Figure S13) and macroscale (cf. the three maps shown in Figure S13).

Figures S14, S15, and S16 prove that CSLN plasmonic substrates meet the criteria #2, #3, and #5 described in Table 1 in the main text (with criteria #1 and #4 addressed in the main manuscript).

Results presented in these three figures confirm sufficient temporal stability, high substrate-to- substrate reproducibility, and SERS activity toward three different analytes, respectively.

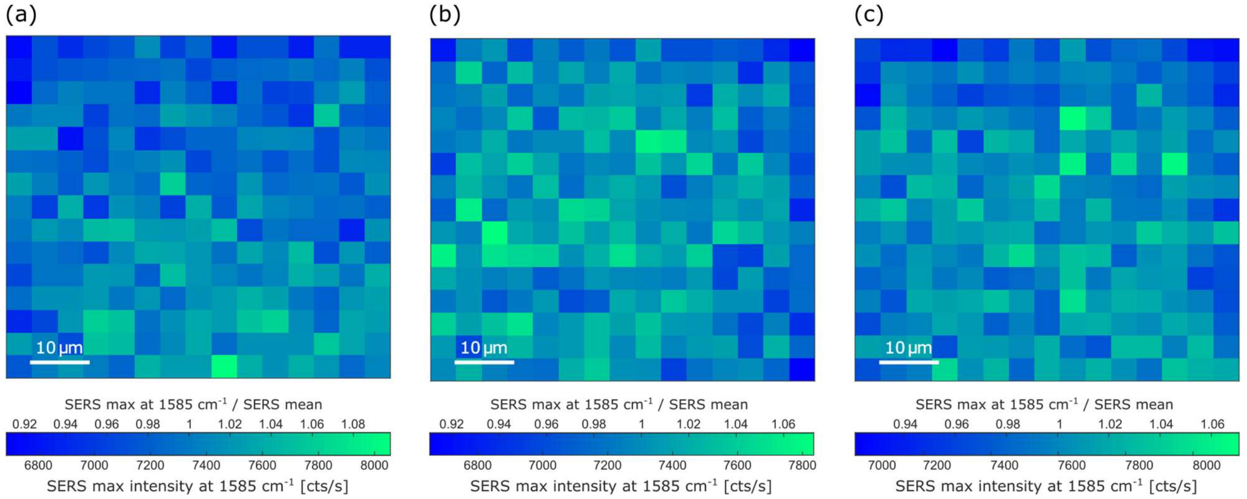

Figure S13: SERS mapping excited with 532 nm laser of three areas (15 × 15 = 225 points for each) at least 1 cm distant from each other for 10^{-4} M pMBA adsorbed on optimized CSLNs (0.2 wt% of 60 nm DNSs, coated with 1.5 nm Ge, 40 nm Ag, and 5 nm Au). Contrast illustrates the spatial distribution of SERS intensity at the maximum of the 1585 cm^{-1} band and the ratio of this intensity at a given point to the mean SERS spectrum from the entire area.

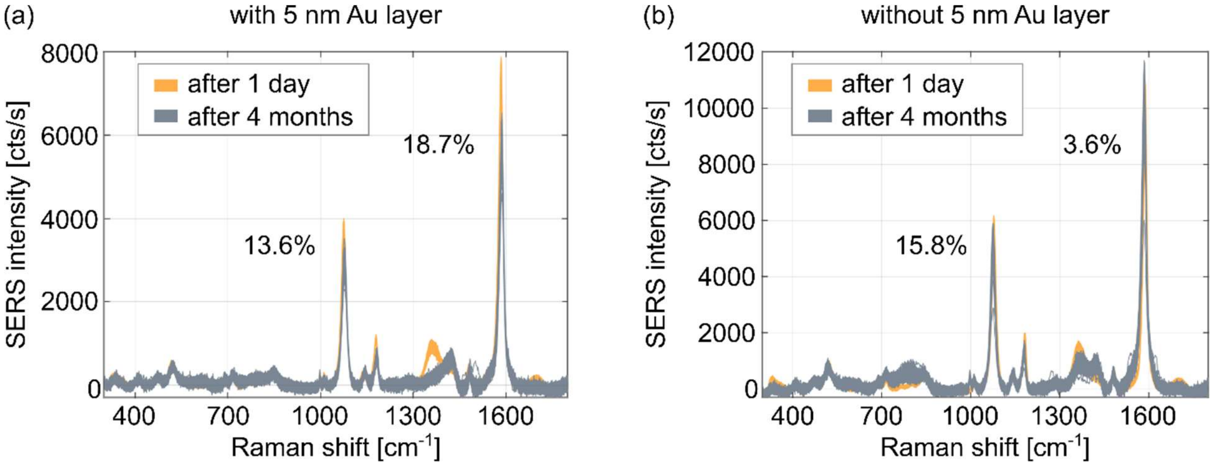

Figure S14: Demonstration of the high-quality temporal stability of CSLN substrates: SERS measurements (excited with 532 nm laser) performed on freshly prepared substrates and 4 months after fabrication. The substrates were fabricated with DNS of *d* = 60 nm and *h* = 40 nm of Ag, either (a) with a 5 nm thick Au layer or (b) without the Au layer. The percentage loss of SERS intensity over 4 months for the two strongest bands in the SERS spectrum of 10^{-4} M pMBA is indicated in the legend.

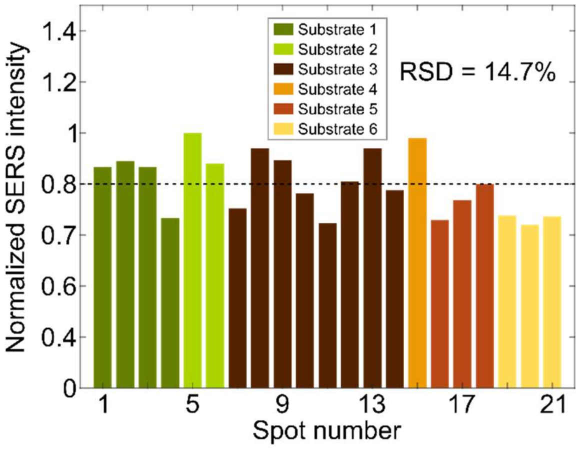

Figure S15: Data confirming sufficient substrate-to-substrate SERS reproducibility of CSLNs. Each bar represents an average signal from 225 SERS spectra (excited with 532 nm laser) of 10^{-4} M pMBA (band at 1585 cm^{-1}) collected for each spot from around 3600 μm^2 rectangular area of 6 substrates fabricated over a period of 5 months. The relative standard deviation (RSD) of the signal across all examined 21 spots is 14.7%. The SERS intensities for each bar are normalized to the highest value.

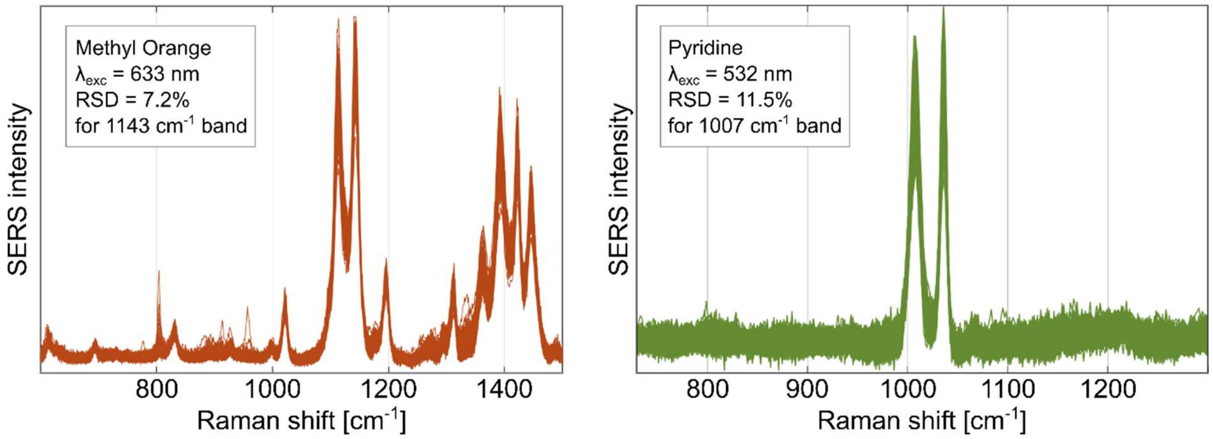

Figure S16: 225 SERS spectra of (a) 10^{-4} M methyl orange solution (MO) and (b) 0.05 M pyridine (Pyr) in 0.1 M KCl solution, both collected for analytes adsorbed on optimized CSLN substrates with *d* = 60 nm coated with and *h* = 40 nm of for Ag and a 5 nm of Au layer. The collection of SERS spectra for Pyr is a part of one of the data sets used to determine the enhancement factor (*EF*). Excitation wavelengths and RSD values of SERS intensity are given in the legend.

Literature

(1) Duan, H. G.; Hu, H. L.; Kumar, K.; Shen, Z. X.; Yang, J. K. W. Direct and Reliable Patterning of Plasmonic Nanostructures with Sub-10-nm Gaps. *Acs Nano* **2011**, *5* (9), 7593-7600.
(2) Xiang, Q.; Li, Z. Q.; Zheng, M. J.; Liu, Q.; Chen, Y. Q.; Yang, L.; Jiang, T.; Duan, H. G. Sensitive SERS detection at the single-particle level based on nanometer-separated mushroom-shaped plasmonic dimers. *Nanotechnology* **2018**, *29* (10).
(3) Wang, X. J.; Zhu, X. P.; Chen, Y. Q.; Zheng, M. J.; Xiang, Q.; Tang, Z. X.; Zhang, G. H.; Duan, H. G. Sensitive Surface-Enhanced Raman Scattering Detection Using On-Demand Postassembled Particle-on-Film Structure. *ACS applied materials & interfaces* **2017**, *9* (36), 31102-31110.
(4) Li, M.; Xun, K. X.; Zhu, X. A.; Liu, D.; Liu, X.; Jin, X. S.; Wu, M. L. Research on AFM tip-related nanofabrication of two-dimensional materials. *Nanotechnol Rev* **2023**, *12* (1).
(5) Ye, Y. T.; Wang, J. Q.; Fang, Z.; Yan, Y. D.; Geng, Y. Q. Periodic Folded Gold Nanostructures with a Sub-10 nm Nanogap for Surface-Enhanced Raman Spectroscopy. *ACS applied materials & interfaces* **2024**, *16* (8), 10450-10458.
(6) Yan, Y. D.; Wang, J. Q.; Geng, Y. Q.; Zhang, G. X. Material removal mechanism of multi-layer metal-film nanomilling. *Cirp Ann-Manuf Techn* **2022**, *71* (1), 61-64.
(7) Zhou, Z. W.; Zhao, Z. Y.; Yu, Y.; Ai, B.; Möhwald, H.; Chiechi, R. C.; Yang, J. K. W.; Zhang, G. From 1D to 3D: Tunable Sub-10 nm Gaps in Large Area Devices. *Advanced Materials* **2016**, *28* (15), 2956-2963.
(8) Xiao, Z. F.; Chen, C.; Xiao, G.; Jiang, Y.; Lu, X. M.; Wang, Y.; Ai, B.; Zhang, G. Customization of Nanogap Arrays Using Stereolithography and Nanoskiving for Surface-Enhanced Raman Scattering. *Acs Appl Nano Mater* **2024**, *7* (23), 27794-27794.
(9) Zhang, P. P.; Wu, J.; Wang, S.; Fang, J. H. Fabrication of triangular Au/Ag nanoparticle arrays with sub-10 nm nanogap controlled by flexible substrate for surface-enhanced Raman scattering. *Nanotechnology* **2023**, *34* (1).
(10) Liu, L.; Zhang, Q.; Lu, Y. S.; Du, W.; Li, B.; Cui, Y. S.; Yuan, C. S.; Zhan, P.; Ge, H. X.; Wang, Z. L.; et al. A high-performance and low cost SERS substrate of plasmonic nanopillars on plastic film fabricated by nanoimprint lithography with AAO template. *Aip Adv* **2017**, *7* (6).
(11) Michota, A.; Bukowska, J. Surface-enhanced Raman scattering (SERS) of 4-mercaptobenzoic acid on silver and gold substrates. *J Raman Spectrosc* **2003**, *34* (1), 21-25.
(12) Capocefalo, A.; Mammucari, D.; Brasili, F.; Fasolato, C.; Bordi, F.; Postorino, P.; Domenici, F. Exploring the Potentiality of a SERS-Active pH Nano-Biosensor. *Front Chem* **2019**, *7*.
(13) Williams, A.; Flynn, K. J.; Xia, Z. D.; Dunstan, P. R. Multivariate spectral analysis of pH SERS probes for improved sensing capabilities. *J Raman Spectrosc* **2016**, *47* (7), 819-827.
(14) Li, R.; Lv, H.; Zhang, X.; Liu, P.; Chen, L.; Cheng, J.; Zhao, B. Vibrational spectroscopy and density functional theory study of 4-mercaptobenzoic acid. *Spectrochimica acta. Part A, Molecular and biomolecular spectroscopy* **2015**, *148*, 369-374.
